# New Strategies and Artificial Intelligence Methods for the Mitigation of Toxigenic Fungi and Mycotoxins in Foods

**DOI:** 10.3390/toxins17050231

**Published:** 2025-05-07

**Authors:** Fernando Mateo, Eva María Mateo, Andrea Tarazona, María Ángeles García-Esparza, José Miguel Soria, Misericordia Jiménez

**Affiliations:** 1 Department of Electronic Engineering, ETSE, (UV), Burjassot, 46100 Valencia, Spain; fernando.mateo@uv.es; 2 Department of Microbiology and Ecology, Faculty of Medicine and Odontology, University of Valencia (UV), 46010 Valencia, Spain; eva.mateo@uv.es; 3 Department of Microbiology and Ecology, Faculty of Biology, (UV), Burjassot, 46100 Valencia, Spain; andrea.tarazona@uv.es; 4 Department of Pharmacy, Cardenal Herrera University-CEU Universities, 46115 Valencia, Spain; maria.garcia2@uchceu.es; 5 Department of Biomedical Sciences, Cardenal Herrera University-CEU Universities, 46115 Valencia, Spain; jose.soria@uchceu.es

**Keywords:** mycotoxins, toxigenic fungi, essential oils, polyphenols, cold plasma, lactic acid bacteria, nanoparticles, magnetic materials, irradiation, machine learning

## Abstract

The proliferation of toxigenic fungi in food and the subsequent production of mycotoxins constitute a significant concern in the fields of public health and consumer protection. This review highlights recent strategies and emerging methods aimed at preventing fungal growth and mycotoxin contamination in food matrices as opposed to traditional approaches such as chemical fungicides, which may leave toxic residues and pose risks to human and animal health as well as the environment. The novel methodologies discussed include the use of plant-derived compounds such as essential oils, classified as Generally Recognized as Safe (GRAS), polyphenols, lactic acid bacteria, cold plasma technologies, nanoparticles (particularly metal nanoparticles such as silver or zinc nanoparticles), magnetic materials, and ionizing radiation. Among these, essential oils, polyphenols, and lactic acid bacteria offer eco-friendly and non-toxic alternatives to conventional fungicides while demonstrating strong antimicrobial and antifungal properties; essential oils and polyphenols also possess antioxidant activity. Cold plasma and ionizing radiation enable rapid, non-thermal, and chemical-free decontamination processes. Nanoparticles and magnetic materials contribute advantages such as enhanced stability, controlled release, and ease of separation. Furthermore, this review explores recent advancements in the application of artificial intelligence, particularly machine learning methods, for the identification and classification of fungal species as well as for predicting the growth of toxigenic fungi and subsequent mycotoxin production in food products and culture media.

## 1. Introduction

This review covers novel strategies and methodologies for managing toxigenic fungi that proliferate in food products and produce mycotoxins. It also includes approaches based on artificial intelligence (AI) for fungal identification, as well as for predicting fungal growth and mycotoxin production. Mycotoxins are secondary metabolites produced by specific species of fungi that contaminate crops and foodstuffs, primarily belonging to the genera *Aspergillus, Fusarium, Penicillium*, *Alternaria,* and *Claviceps*. The main mycotoxins and the corresponding fungal producers are summarized in Table 1. They include aflatoxins (AFs), particularly the aflatoxins B1 (AFB1), B2 (AFB2), G1 (AFG1), and G2 (AFG2), produced by *Aspergillus flavus*, *A. parasiticus*, and other less known species; ochratoxins, particularly ochratoxin A (OTA), synthesized by certain species of *Aspergillus* and *Penicillium;* patulin (PAT) produced mainly by *P. expansum*; type-A trichothecenes (e.g., T-2 and HT-2 toxins, diacetoxyscirpenol (DAS)), and type-B trichothecenes such as deoxynivalenol (DON), nivalenol (NIV), 3- and 15-acetyldeoxynivalenol (3- and 15-ADON); zearalenone (ZEA); fumonisins (FUMs), mainly fumonisin B1 (FB1) and B2 (FB2) produced by *Fusarium* spp.; and citrinin (CIT). Ergot alkaloids are produced by *Claviceps* spp., while *Alternaria* spp. are responsible for secondary metabolites including altenuene, alternariol, alternariol methyl ethers, altertoxins, and tenuazonic acids [1]. Other emerging mycotoxins such as fusaproliferin, moniliformin, beauvericin, and enniatins primarily biosynthesized by *Fusarium* spp. are attracting increasing interest among the scientific community. Mycotoxins can cause serious diseases in humans and animals that have been clearly demonstrated [1,2].

Although several hundred mycotoxins are known, only a few of these are regulated in the European Union (EU) in a variety of foods [3,4,5]. These mycotoxins are present in certain foods and beverages that are considered more susceptible to contamination because they are raw materials or food derivatives of raw materials. These materials usually contain the fungi that produce them [6,7,8]. These key mycotoxins are the following: AFB1, total AFs (the sum of AFB1, AFB2, AFG1, and AFG2), AFM1, OTA, the sum of FB1 and FB2, PAT, DON, ZEA, CIT, the sum of T-2 and HT-2 toxins, and ergot alkaloids. In addition, the content of ergot sclerotia in non-transformed cereal grains is also regulated. AFM1 is produced as a result of AF metabolism in animal organism and may be present in dairy products [2].

Some approaches developed to control food contamination by mycotoxins and other contaminants throughout the food supply chain, from farm to fork, are known as Hazard Analysis and Critical Control Point (HACCP) procedures. These procedures are implemented to address physical, chemical, and biological hazards as a proactive approach to prevent contamination, rather than relying solely on post-inspection measures to ensure food safety. HACCP procedures can be categorized into preharvest and postharvest strategies [2,9]. In the context of this review, preharvest strategies focus on preventing mycotoxin production in the field. They include the implementation of good agricultural practices, such as proper cropping systems, the use of tolerant cultivars, the application of fungicides, and biocontrol protocols. Postharvest involves identifying critical control points during harvesting, drying, and storage, which are essential for the development of effective detoxification strategies [10].

Numerous studies have explored alternative and eco-friendly strategies to manage fungal contamination and mycotoxin biosynthesis. A recent comprehensive review conducted by Hamad et al. [11] examined the use of cold atmospheric plasma, polyphenols, nanoparticles, magnetic materials, and natural essential oils for mycotoxin control in food systems. Their work provided a valuable synthesis of emerging technological strategies and their mechanisms of action, offering a significant reference point for researchers and practitioners in the field. Building upon this foundation, the present review aims to broaden the perspective by incorporating additional studies, different viewpoints, and strategies not covered in the study of [11]. Specifically, this includes the use of lactic acid bacteria, irradiation, techniques primarily ionizing radiation, and the application of artificial intelligence-based tools. Therefore, the present review complements the overview exposed in the study of [11] by providing a more comprehensive perspective on innovative approaches to combat against toxigenic fungi and their mycotoxins. By integrating these additional strategies, this review aims to contribute to the overarching goal of mitigating or eliminating mycotoxin accumulation, ensuring food safety for both humans and animals while maintaining environmental sustainability and cost-effectiveness. The recent rise in artificial intelligence (AI) has also driven the development of machine learning (ML) and deep learning (DL) methods to predict fungal growth rates and mycotoxin production in food primarily under laboratory conditions. The long-term goal is to apply these techniques in the field by creating accurate predictive models to support crop protection.

In the following sections, these emerging strategies or methodologies will be discussed, with a focus on their advantages and limitations as antifungal agents for food protection

## 2. Essential Oils

### 2.1. Concept of Essential Oils

Essential oils (EOs) are complex mixtures of generally volatile low-molecular-weight compounds derived from aromatic plant materials such as buds, flowers, seeds, leaves, twigs, barks, fruits, and roots. In the natural environment, EO components often serve as part of the plant’s innate defense mechanisms against pathogens. These substances are commonly extracted through methods such as hydrodistillation, microwave-assisted dry distillation, or steam distillation methods [12].

EOs are soluble in alcohol, ether, and fixed oils but insoluble in water. They are classified are volatile oils and are generally liquid and colorless at room temperature. EOs possess a characteristic odor and typically have a density lower than that of water, with exceptions including cinnamon, sassafras, and vetiver oils [13]. These compounds are synthesized in the cytoplasm and are usually present as minute droplets located between plant cells.

In recent years, there has been increasing societal awareness regarding the importance of a “green, safe, and clean” environment. This shift has coincided with a growing demand for “green consumerism”, encouraging the development of food products free from synthetic preservatives [12].

### 2.2. Composition

EOs have demonstrated significant potential as food preservatives, owing to their broad-spectrum antimicrobial properties. However, their complex chemical composition (comprising over 500 individual compounds) necessitates a thorough understanding of their key constituents, antibacterial and antifungal mechanisms of action, and target organisms to ensure effective and targeted application in food preservation [12].

The primary bioactive constituents responsible for the antimicrobial properties of EOs can be isolated and utilized individually, thus avoiding the need to apply the complete mixture. These components predominantly include terpenes, terpenoids, and phenylpropanoids. Terpenes are hydrocarbons made from isoprene (2-methyl-1,3-butadiene) (C_5_H_8_) units linked one to another, and they typically occur in EOs as monoterpenes (C_10_H_16_) or sesquiterpenes (C_15_H_24_). Although diterpenes (C_20_H_32_), triterpenes (C_30_H_48_), and tetraterpenes (C_40_H_64_) may also be present, they are generally found in smaller quantities [14].

Monoterpenes can be acyclic, monocyclic, or bicyclic. Representative compounds include camphene, α-pinene, β-pinene, sabinene, limonene, p-cymene, α-terpinene, citronelle, ocymene, and β-myrcene. Similarly, sesquiterpenes can be acyclic (e.g., farnesene, farnesane), monocyclic (e.g., α-zingiberene, α-humulene, germacrene D), or bicyclic (e.g., selinene, chamigrane, eremophilane, β-caryophyllene).

Terpenoids are oxygenated derivatives of terpenes, with various functional and oxidized methyl groups moved or removed at various positions. Terpenoids can be classified based on the presence of specific functional groups, namely, alcohols, esters, aldehydes, ketones, ethers, and epoxides. Common monoterpenoids with hydroxyl groups include menthol, geraniol, nerol, thymol, carvacrol, geraniol, linalool, citronellol, terpineol, myrtenol, carveol, and dihydrocarveol. Other derivatives include esters (linalyl acetate, geranyl acetate), aldehydes (citronellal, myrtenal), and ketones (carvone, dihydrocarvone) [12].

**Figure 1:**
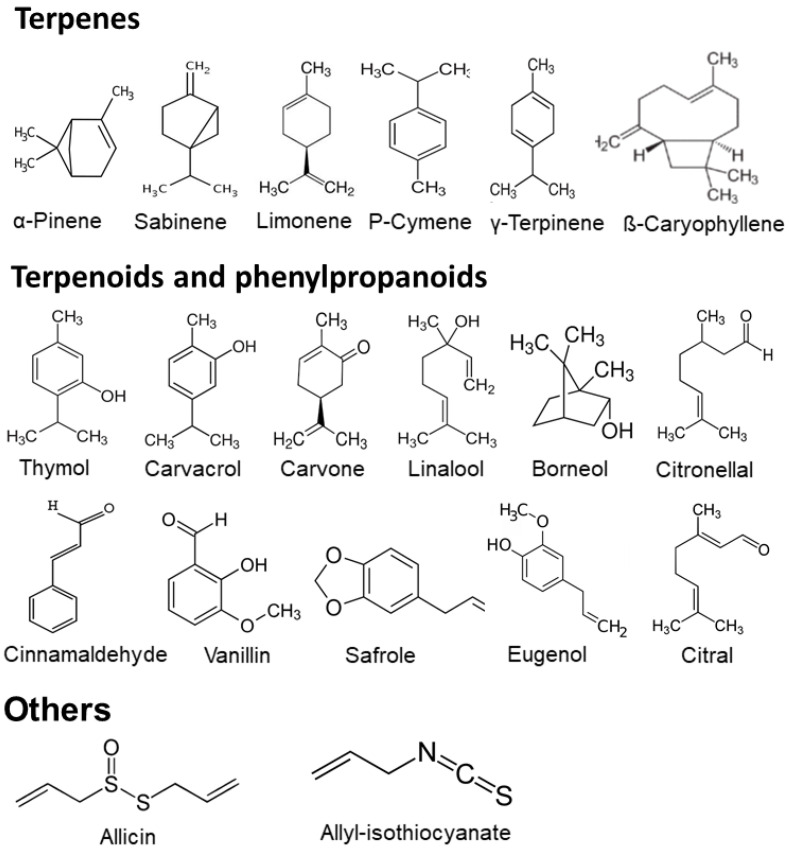
Structural formulas of some of the major constituents of the EOs.

In addition to terpenes and terpenoids, EOs also contain other bioactive compounds such as phenylpropanoids (e.g., eugenol, isoeugenol, trans-cinnamaldehyde), and sulfur-containing compounds (e.g., allycin, allyl isothiocyanate). The structural configurations of several important EO components are presented in Figure 1.

### 2.3. Application as Food Preservatives

Since the Middle Ages, various EOs have been employed for their fungicidal, bactericidal, insecticidal, and antiparasitic properties. In recent years, there has been a growing body of the scientific literature highlighting their application as antifungal agents in food preservation. EOs offer several advantages: they are volatile, can be applied as fumigants, are environmentally friendly, and are safe for human health. In the United States, many EO-based formulations are classified as “Generally Recognized as Safe” (GRAS) by the Food and Drug Administration (FDA) and the Environmental Protection Agency (EPA) [15].

EO-based preservatives have been developed by industries and are commercially available [16]. These products represent a promising alternative to synthetic chemical fungicides traditionally used in agriculture for crop and food protection. Their efficacy is attributed to a dual mode of action, simultaneously inhibiting fungal growth and suppressing mycotoxin production [17,18].

On a global scale, the abuse of synthetic fungicides has contributed to the emergence of resistant microbial strains. This has necessitated the use of higher fungicide concentrations on agricultural lands, leading to increased production costs, elevated risks to human health, and environmental contamination due to the accumulation of chemical residues in water, soil, and food products [19].

### 2.4. Mechanisms of Antifungal Action

The antifungal activity of EOs is mainly attributed to their lipophilic and low-molecular-weight components, which can readily penetrate the fungal plasma membrane. This interaction disrupts membrane permeability and the osmotic balance of the cell, triggering proton efflux and subsequent intracellular alterations that ultimately lead to cell death or the inhibition of sporulation and germination in food spoilage fungi [20,21]. Adverse effects may be categorized as primary, secondary, or tertiary in view of the degree of implication of the EOs in such effects.

EOs can disrupt and penetrate the fungal cell wall and the plasma membrane (primary effects) by interacting with its lipid components, leading to increased permeability and leakage of vital intracellular contents. Then, they can interact with enzyme active sites, such as that of chitin synthase, thereby inhibiting chitin polymerization. Such inhibition (a secondary effect) affects multiple processes including cell wall maturation, septum formation, and bud ring development, ultimately compromising cell division and growth as tertiary effects [22]. A similar disruption in β-glucan synthesis has also been reported. The loss of essential ions and intracellular compounds contributes to cell death [23].

This ion leakage, involving Ca^2^⁺, Mg^2^⁺, and K⁺, has been confirmed by atomic absorption spectrophotometry [24]. Studies have demonstrated that specific EO constituents, such as thymol, carvacrol, and eugenol, interact with fungal cell membranes, altering proton (H⁺) and K⁺ gradients and facilitating the leakage of vital intracellular materials. This leads to water stress, a decrease in intracellular ATP levels, and cell death [20].

Additionally, EOs can reduce ergosterol content (a key sterol in fungal plasma membranes that is absent in plants) within the plasma membrane, in a dose-dependent manner [25]. This is a primary effect that has been reported, for example, in maize seeds inoculated with *Fusarium graminearum* and *F. culmorum* [26]. The observed decline in ergosterol levels is suggestive of a decrease in fungal growth. The depletion of ergosterol in fungal membranes compromises membrane integrity, resulting in osmotic imbalance and metabolic instability, thereby impeding fungal growth, reproduction, and pathogenicity [27,28,29].

EOs or their components affect the mitochondrial membrane (a primary effect) and may also inhibit mitochondrial enzymes, such as malate dehydrogenase, which is essential for cellular metabolism in fungi [25]. As a consequence, this inhibition leads to a significant reduction in intracellular ATP levels [29,30]. In *A. flavus*, EO-induced mitochondrial disruption was found to block the synthesis of acetyl-CoA, the primary precursor for AF biosynthesis, effectively inhibiting AFB1 production [20]. In *A. flavus* treated with *Perilla frutescens*, EO additional effects have been observed, including the accumulation of reactive oxygen species (ROS), the leakage of intracellular macromolecules (e.g., proteins), the degradation of some amino acids and inhibition of key enzymes such as Ca^2+^/Mg^2+^-ATPase and Na^+^/K^+^-ATPase, which are secondary effects of the EO activity. These changes contribute to ROS accumulation and hence oxidative stress, which adversely affect the DNA, and may induce apoptosis [29,31]. It has been reported that the EOs have secondary effects on quorum-sensing mechanisms, hindering the ability of fungi to form biofilms and coordinate activities essential for infection and survival [32].

**Figure 2:**
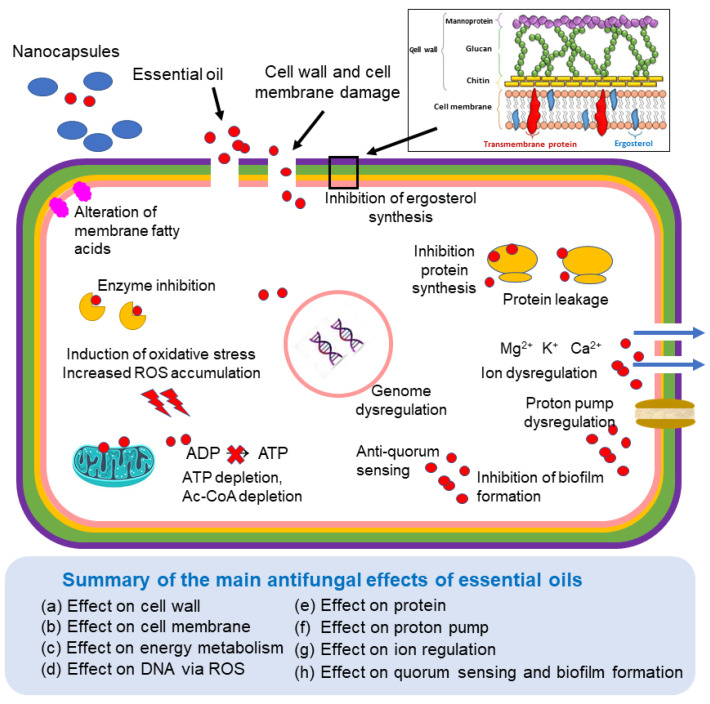
Some effects of the essential oils in the fungal cell. Blue arrows mean efflux of ions. Cell wall detail adapted from [43].

EOs are complex mixtures, and the antifungal activity of individual components can vary significantly. Their effectiveness is often associated with specific functional groups. Phenolic compounds (e.g., eugenol, carvacrol, chavicol, and 4-allyl-2-6-dimethoxyphenol) exhibit the highest antifungal potency, followed by acids such as cinnamic and hydrocinnamic acids. The general order of antifungal activity by chemical functionality is as follows: phenols > cinnamic aldehydes > alcohols > aldehydes ≥ ketones > ethers > hydrocarbons [33]. Nonetheless, the precise mechanisms underlying the antifungal effects of EO constituents remain incompletely understood. Figure 2 illustrates some of the cellular targets and effects of EOs in the fungal cell.

Due to their inherent antimicrobial properties, EOs have attracted significant interest in the food industry and in the field of active packaging, particularly in the development of active packaging systems aimed at extending shelf life and enhancing food safety. Their applications have been explored for the preservation of various food products, including fresh fruits, cereal grains, meat products, and ready-to-eat salads [34,35,36,37,38,39].

However, several limitations hinder the widespread adoption of EOs in food systems. These include the high cost associated with the extraction of EO components in bulk, their limited chemical stability, and the potential for undesirable sensory effect, such as strong odors or flavors that can negatively impact consumer acceptance of the final product. In response, innovative approaches have been developed to enhance the applicability and efficacy of EOs. These innovations include the incorporation of EOs or their components into biodegradable films and their encapsulation within micro- or nano-sized carriers to improve stability, control release, and reduce sensory impact [40,41,42].

While a substantial body of research has focused on the antibacterial properties of EOs across diverse food matrices, relatively few studies have addressed their effectiveness in controlling toxigenic fungi and associated mycotoxin contamination [44,45,46]. This review highlights the antifungal mechanisms of action of EOs and their potential as natural, eco-friendly alternatives to synthetic preservatives for the prevention of fungal spoilage and mycotoxin production in food systems.

### 2.5. Application of EOs Against Toxigenic Fungi

A considerable number of EOs have been shown to possess fungicidal properties and the ability to reduce mycotoxin production [21]. The antifungal and antiaflatoxinogenic activities of EOs from the leaves of *Cymbopogon schoenanthus*, *C. citratus*, and *C. nardus* (lemongrass leaf oil), as well as their pairwise combinations, were evaluated in potato dextrose agar (PDA) medium. Among these, *C. nardus* exhibited the strongest activity against *Aspergillus flavus* and *A. parasiticus*, with synergistic effects observed in mixtures of two EO species [47]. AFB1 production was inhibited at a concentration of 1 µL/mL, while AFB2 and AFG2 production was inhibited at 0.5 µL/mL.

The antifungal, antiaflatoxigenic, and antioxidant properties of EOs from *Mentha pulegium*, *Myrtus communis*, and *Mentha piperita* were evaluated against *A. flavus*. The minimum inhibitory concentration (MIC) of *M. pulegium* and *M. communis* EOs was 4.00 μL/mL, while the MIC of *M. piperita* was 3.50 μL/mL. The 50% inhibitory concentration (IC_50_) values ranged between 3.27 and 4.31 μL/mL [48].

Sonker et al. [49] investigated the antifungal activity of *C. citratus* EO against *Aspergillus ochraceus*, *A. niger*, and *A. flavus*. The MIC was 0.29 µL/mL for all tested fungi, while the minimal fungicidal concentration (MFC) was 0.57 µL/mL for *A. ochraceus* and 0.64 µL/mL for both *A. niger* and *A. flavus*. The complete inhibition of mycelial growth was achieved with all three fungi. Additionally, ochratoxin A production was suppressed at a concentration of 0.8 µL/mL. Lemongrass EO from *C. citratus* prepared by a microfluidization process showed inhibition effects against toxigenic strains of fungi belonging to the *Fusarium* and *Aspergillus* genera and a decrease in mycotoxin production [50].

In the same context, ginger EO exhibited noteworthy antifungal activity against *A. flavus*, with MIC and MFC values of 150 µg/mL. A dose-dependent reduction in mycelial growth was observed, along with suppression of AF biosynthesis via antioxidant mechanisms. EO activity led to decreased fungal biomass, consequently reducing AF production [51].

Abbaszadeh et al. [52] assayed the antifungal activity of thymol, carvacrol, eugenol, and menthol against eleven food-relevant fungi (*A. niger*, *A. fumigatus*, *A. flavus*, *A. ochraceus*, *Alternaria alternata*, *B. cinerea*, *Cladosporium* spp., *P. citrinum*, *P. chrysogenum*, *F. oxysporum*, and *Rhizopus oryzae*). Carvacrol exhibited the highest efficacy, particularly against *Cladosporium* spp. and *Aspergillus* spp. In vitro studies have further confirmed the high antifungal activity of thymol and carvacrol against *F. oxysporum*, *F. verticillioides*, *Penicillium brevicompactum*, *P. expansum*, *A. flavus*, and *A. fumigatus* [53].

Xiang et al. [54] examined the antifungal potential of eleven EOs against *A. flavus* by measuring the mycelial diameter on PDA plates. The results of the vapor test revealed that cinnamon EO had the strongest antifungal activity, completely inhibiting mycelial growth at 1 µL/disk. Oregano and lemongrass EOs were moderately effective, while basil, clove, citronella, *Litsea cubeba*, and thyme showed weaker inhibition. *Artemisia* (mugwort), peppermint, and rosemary EOs exhibited the least efficacy. A mixture of cinnamon, oregano, and lemongrass EOs (COL) showed enhanced antifungal activity, as evidenced by low MIC values under vapor conditions. A chemical analysis of the composite EOs of cinnamon, oregano, and lemongrass (COL-CEO) identified (*Z*)-citral (33.44%), (*E*)-citral (32.88%), and carvacrol (19.84%) as the major constituents, followed by limonene (4.29%) and cinnamaldehyde (3.76%). COL-CEO also significantly inhibited AFB1 production by *A. flavus*.

A nanoemulsion composed of salted cellulose nanocrystals and Tween 8, loaded with lemongrass EO, demonstrated greater antifungal activity against *A. flavus* mycelial growth than the pure EO alone [55] in agreement with a previous study [50].

The antifungal and antiaflatoxigenic activity of rosemary (*Rosmarinus officinalis*) EO against *A. flavus* has also been demonstrated [56]. Another study investigated the effects of EOs from *Origanum vulgare* an *Cinnamomum zeylanicum*, or their major active constituents, carvacrol and cinnamaldehyde, respectively, incorporated into ethylene-vinyl alcohol copolymer films (EVOH) on the growth of *A. flavus* and *A. parasiticus* and AF production in maize grains under various a_w_ and temperature conditions [57]. The assays, performed the in vitro and in vapor phase, revealed that EO type, fungal species, temperature, and a_w_ significantly influenced the effective dose 50 (ED_50_). The antifungal efficacy of the films ranked as follows: EVOH-cinnamaldehyde > EVOH-carvacrol > EVOH-oregano > EVOH-cinnamon. These results underscore the potential of EVOH-EO films as promising tools for the control of fungal growth and AF contamination in stored maize grains.

When applied directly to solid culture media, the same EOs and constituents also demonstrated antifungal and anti-aflatoxin activity, though with lower efficacy than vapor-phase bioactive films [58].

In another study, EVOH films incorporating citral, cinnamaldehyde, isoeugenol, or linalool EOs were examined in vitro against *Fusarium sporotrichioides* on oat grains. These films were evaluated for their ability to inhibit fungal growth and the production of T-2 and HT-2 toxins. EVOH–citral showed the highest effectiveness, followed by EVOH–cinnamaldehyde, while EVOH–linalool was the least effective. The inhibition of mycotoxin production was directly correlated with reduced or completely halted fungal growth [59].

Garlic EO has also shown antifungal effects against *A. parasiticus*, *F. verticillioides*, and *F. graminearum* [60]. Notably, the concentration of garlic EO required to suppress mycotoxin production was four times lower than the concentration needed to inhibit fungal growth.

Following the designation of EOs as GRAS, interest in their use for food preservation has grown considerably. This interest has catalyzed the development of novel delivery systems, including encapsulation techniques [61] and the application of EOs in edible films or coatings [62]. The feasibility of using microencapsulation methods to incorporate EOs into food matrices has also been demonstrated [63].

### 2.6. Regulations of Essential Oil Applications in Food Systems

EOs have found widespread applications in various industries, including cosmetics, food, and pharmaceuticals. In the United States, the regulation of EOs falls under the jurisdiction of the FDA, specifically Section 21 CFR 182.20 of the Code of Federal Regulations (CFR) 2022 [64], and is also aligned with California Proposition 65 and other relevant regulatory frameworks.

In Europe, the European Commission (EC) has approved a wide range of EO compounds for use as flavoring agents in different categories of food products. EOs derived from mustard, oregano, clove, cinnamon, nutmeg, thyme, basil, rosemary, and lavender are recognized as GRAS. In 2008, the EC published a comprehensive list of approved flavoring substances, which is periodically updated. A number of flavoring compounds have been registered as safe for human consumption. These include limonene, linalool, β-caryophyllene, pinene, thymol, carvacrol, carvone, eugenol, isoeugenol, vanillin, citral, citronellal, cinnamaldehyde, menthol and lavandulol, all of which are also recognized as GRAS compounds [65,66].

To ensure consumer safety, regulatory agencies have established limits on the acceptable daily intake (ADI) of EOs and their constituent compounds in food applications [66]. Nevertheless, despite these safety regulations, some EOs have been reported to trigger allergic reactions in sensitive individuals. Furthermore, the ingestion of high doses or prolonged topical use of certain EOs has been associated with adverse health effects, including oral toxicity and contact dermatitis [67].

Therefore, achieving an optimal balance between efficacy and safety is critical when incorporating EOs into food products. Appropriate dosage and delivery systems must be carefully evaluated to ensure both functional effectiveness and consumer safety.

## 3. Phenolic Compounds

### 3.1. Concept and Classification

**Figure 3:**
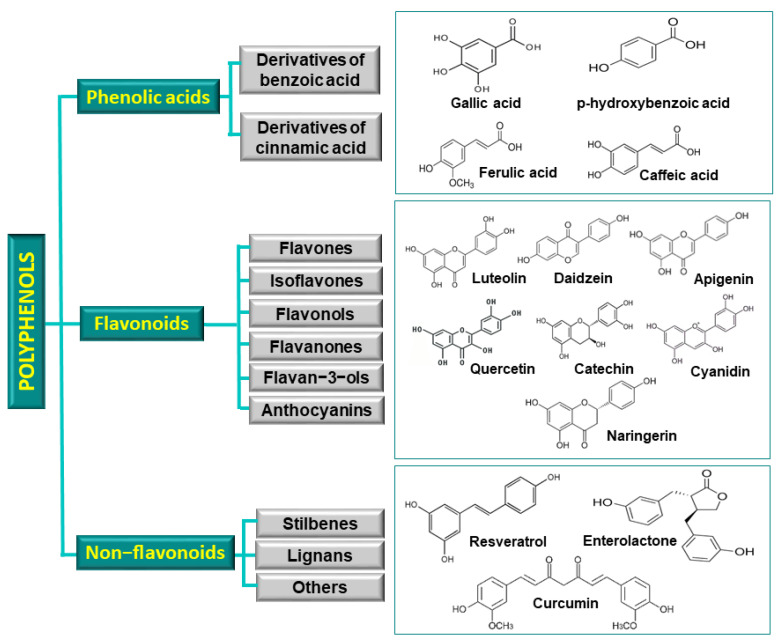
Classification of polyphenols.

Polyphenols are secondary metabolites characterized by the presence of one or more aromatic rings bearing two or more hydroxyl groups. These compounds are widely distributed in plant tissues, including roots, stems, leaves, flowers, fruits, and seeds. Polyphenols include a diverse group of molecules such as phenolic acids, flavonoids, and non-flavonoid compounds such as stilbenes, lignans, and other compounds, including tannins (Figure 3).

Among these, phenolic acids are the simplest in terms of chemical structure. They are generally categorized into two main groups based on their core structure: benzoic acid and derivatives of cinnamic acid. Examples of benzoic acid derivatives include gallic, p-hydroxybenzoic, protocatechuic acid, and syringic acid. Cinnamic acid derivatives include chlorogenic, ferulic, vanillic, caffeic, *p*-coumaric, and sinapic acids. These compounds feature a six-carbon aromatic phenol group linked to a three-carbon propene side chain, typical of cinnamic acid. They are synthesized through the shikimate pathway, initiated by the deamination of phenylalanine [68,69].

Flavonoids constitute the most abundant class of naturally occurring phenolic compounds, present in plant tissues as glycosides, aglycones, and methylated derivatives [70]. This class encompasses over 9000 compounds, many of which exhibit antimicrobial properties, among other biological activities. Structurally, flavonoids are characterized by two phenolic rings connected by a three-carbon bridge, forming a heterocyclic pyran or pyrone ring [71]. The degree of oxidation and saturation of the central heterocyclic ring allows for their classification into distinct subclasses, including flavones, flavanones, flavanols, isoflavones, flavan-3-ols, and anthocyanins [72,73,74].

Biosynthetically, flavonoids are specialized metabolites derived from the phenylalanine and acetate metabolic pathways [10]. Their synthesis in plants is often induced by environmental stressors such as pathogen attack, nutrient deficiencies, or increased light exposure [74].

Among non-flavonoid polyphenols, stilbenes are notable for their 1,2-diphenylethylene (C6–C2–C6) backbone, comprising an ethylene bridge flanked by two hydroxylated benzene rings. Resveratrol serves as a prominent example of this group. Lignans are another class of non-flavonoid polyphenols, categorized into lignans, neolignans, and oxyneolignans based on the nature of the carbon–carbon bond and the presence of an oxygen bridge connecting two phenylpropane units [75]. These compounds exhibit considerable structural diversity and are distributed across various plant tissues, including roots, stems, leaves, and seeds.

Tannins represent high-molecular-weight polyphenols, ranging from 500 Da to 30,000 Da, and are widespread throughout the plant kingdom. They are primarily classified into two groups: condensed tannins and hydrolysable tannins [76].

### 3.2. Properties and Activities

Phenolic compounds exhibit significant antioxidant properties, enabling them to inhibit free radical reactions. This antioxidant activity aids plants in mitigating pathogenic infections and safeguarding vital tissues from the deleterious effects of reactive oxygen species (ROS) [74]. The efficacy of these compounds as antioxidants is largely influenced by their molecular structure, particularly the number and position of hydroxyl groups and the presence of unsaturated bonds.

Natural phenolic compounds are increasingly investigated as potential fungicidal agents against toxigenic fungi, particularly *Fusarium* species. Certain phenolic compounds are reported as strong inhibitors of mycotoxin production by these fungi [77]. Natural acids, including gallic, caffeic, and chlorogenic acids possess inhibitory activity against the production of various mycotoxins, such as FB1, OTA, and type B trichothecenes [10].

### 3.3. Mechanism of Antifungal Action

A variety of mechanisms appear to contribute to the inhibition of mycotoxin production, including structural alterations of the fungal cell membrane and cell wall, the downregulation of genes associated with mycotoxin biosynthesis, and the inhibition of enzyme activities involved in oxidative stress regulation [10]. Enzyme inhibition may disrupt metabolic pathways, while membrane interference can lead to cell death.

The antifungal mechanism of action of tea polyphenols, assayed in vitro against *Rhizopus stolonifer*, a postharvest fruit pathogen, may involve both direct damage to mycelia and spores and the indirect induction of defensive enzyme activity [78]. These compounds have been shown to stimulate the production of hydrogen peroxide (H_2_O_2_), which promotes membrane lipid peroxidation. This process increases membrane permeability, leading to the leakage of K^+^, soluble protein, and sugars. Consequently, the induction of ROS production appears to play a central role in the antifungal activity of tea polyphenols and saponins [79].

Ginger extract, rich in phenolic compounds, has demonstrated protective effects against AFB1-induced damage in vitro by suppressing intracellular ROS production, which is associated with DNA strand breaks. Moreover, in vivo, ginger extract reduces lipid peroxidation [80]. Similarly, a phenolic extract of palm kernel cake, containing gallic acid, vanillic acid, caffeic acid, syringic acid, ferulic acid, pyrogallol, epicatechin, and catechin, exhibited protective effects against AFB1-induced toxicity by reducing lipid peroxidation and enhancing antioxidant enzyme production [81]. Additionally, gallic acid has been reported to alleviate liver and kidney dysfunction caused by AFB1 exposure [82].

### 3.4. Application of Polyphenols as Antifungal Agents

A wide range of phenolic compounds (phenolic acids, flavonoids, stilbenes, lignans, etc.) has been examined in vitro for their ability to inhibit the synthesis of major mycotoxins commonly found in food and feed products [10].

Badr et al. [70] investigated the antifungal activity of an extract derived from mandarin (*Citrus sinensis*) by-products against *Fusarium* species. The extract contained phenolic acids, flavonoids and terpenes. While the crude extract reduced *Fusarium* growth in vitro, its efficacy was lower than that of hesperidin applied directly to the fungal medium. The extract also reduced mycotoxin production by *Fusarium* spp.; however, due to the complex mixture of bioactive compounds, the observed activity cannot be solely attributed to phenolic constituents.

Phenolic extracts obtained from cereal cultivars experimentally inoculated with *Fusarium* fungi exhibited antifungal activity against *F. culmorum* (NIV chemotype), *F. culmorum* (3-ADON producer), *F. graminearum* (3-ADON producer), *F. graminearum* (NIV chemotype) and *F. langsethiae* [69]. In contrast, extracts derived from non-inoculated cereals did not demonstrate any antifungal activity.

The in vitro effects of four phenolic acids (caffeic, chlorogenic, ferulic, and p-coumaric) on fungal biomass and the production of T-2 and HT-2 toxins by *F. langsethiae* and *F. sporotrichioides* were studied [83]. The results demonstrated that *F. langsethiae* was more sensitive to phenolic acids, with chlorogenic and ferulic acids significantly reducing toxin production. In contrast, *F. sporotrichioides* was only affected by ferulic acid. Furthermore, *F. sporotrichioides* produced higher levels of T-2 and HT-2 toxins than *F. langsethiae*, regardless of treatment.

Naturally occurring phenolic compounds, including phenolic acids, flavonoids, stilbenes, and lignans, have been shown to effectively inhibit the in vitro production of AF, FUM, trichothecenes, ZEA, OTA, and PAT. Bioactive compounds isolated from *Salvia* spp. have demonstrated antifungal activity against various *Fusarium* species, including *F. tricinctum, F. sporotrichioides,* and *F. oxysporum* f. sp. *radicis-lycopersici* [84,85].

Further research assessed the antifungal activity of plant extracts from sage *(Achillea millefolium* L.), tansy (*Tanacetum vulgare* L.), sage (*Salvia officinalis* L.) and wormwood (*Artemisia absinthium* L.) against *Fusarium* spp. The results demonstrated a concentration-dependent effect on fungal growth. with the most significant reductions observed at 20% extract concentration. Among the tested plant, sage and tansy extracts exhibited the strongest antifungal activity, particularly against *F. culmorum*, *F. avenaceum*, and *F. sporotrichioides*. In contrast, wormwood extracts were the least effective [86].

Certain phenolic compounds, such as caffeic acid, vanillic acid, chlorophorin, and makenianin, have shown potent inhibitory effects against *F. verticillioides* achieving over 90% reduction in FB1 levels [87]. Phenolic extracts do not always significantly affect fungal growth. However, they have been shown to be effective in inhibiting mycotoxin synthesis. For instance, methanol extracts from cultivated mushrooms did not significantly inhibit the growth of *F. verticillioides* yet they suppressed FUM synthesis by up to 79%, 92%, and 69% for FB1, FB2, and FB3, respectively [88]. Similarly, ferulic acid had no impact on fungal biomass but reduced FB1 production by 90% [89].

Although phenolic compounds are generally not effective against OTA contamination in food, supplementation with these compounds during exposure to the toxin has shown potential in mitigating OTA-induced damage [90]. Most phenolic acids evaluated for antifungal activity against *F. oxysporum*, *F. verticillioides*, *P. brevicompactum*, *P. expansum*, *A. flavus*, and *A. fumigatus* exhibited limited efficacy compared to thymol and carvacrol [53].

Three flavonoids (apigenin, luteolin, and quercetin) were applied to *A. flavus* NRRL 3357 to evaluate their morphological and biochemical effects. All three compounds, applied at 1 ng/µL, induced structural alterations in the mycelial network. Quercetin treatment led to severe mycelial collapse, resulting in flattened fibers. Apigenin and luteolin caused peeling of the outer cell wall layer; in addition, apigenin induced fiber detachment and the formation of web-like structures, while luteolin treatment caused vesicle-like surface features. These flavonoid treatments were also associated with increased ergosterol content and elevated expression of related genes [91].

### 3.5. Challenges in Practical Application and Regulatory Considerations

The routine application of phenolic compounds is hindered by several limitations, including their instability under processing and storage conditions, such as exposure to heat, moisture, and light, as well as their limited solubility and susceptibility to oxidative degradation in aqueous environments [92]. Moreover, many phenolic compounds exhibit an undesirable bitter or astringent taste, which restricts their incorporation into food and pharmaceutical formulations.

Although polyphenols are commonly consumed through fruits and vegetables, in Europe, isolated polyphenols and plant extracts may be classified as novel foods or food additives. The European Food Safety Authority (EFSA) requires a comprehensive scientific evaluation prior to their approval, necessitating robust evidence to establish their safety. Current European regulations concerning novel foods and food additives are complex and multifaceted, reflecting stringent standards aimed at ensuring food safety. To date, only a limited number of novel food products containing polyphenols have received approval within the European Union (EU), and these approvals often include restrictions on permissible doses [93].

Furthermore, current regulations in both the EU and the United States do not authorize the use of plant extracts with high concentrations of phenolic compounds for the specific purpose of mycotoxin control [10]. In summary, while polyphenols are not uniformly regulated as food additives, their use remains subject to broader food safety frameworks in both the EU and the USA.

## 4. Cold Plasma Technology (CPT)

### 4.1. Concept and Types of CP

Cold plasma (CP) also referred to as cold plasma technology (CPT) is an advanced non-thermal processing technique with growing relevance in the food industry. It possesses the capacity to inactivate microorganisms and degrade chemical contaminants, including mycotoxins, thereby offering a promising approach to enhancing food safety [94].

Plasma is a partially ionized gas comprising a complex mixture of reactive neutral species, energetic charged particles, ions, electrons, ultraviolet (UV) photons, and strong electric fields. These constituents interact simultaneously and synergistically with food matrices, contributing to both microbial inactivation and chemical detoxification [95,96]. Plasma is produced when a gas is exposed to sufficient energy, typically via heat or an electric field, resulting in ionization and the formation of plasma. While plasma is commonly associated with high temperatures (e.g., solar plasma), cold plasma is a non-thermal variant that operates at or near room temperature and atmospheric pressure, without achieving thermodynamic equilibrium. Its rich composition of reactive chemical species renders it suitable for a range of applications, particularly in pathogen control [94].

This emerging technology has demonstrated significant potential across multiple sectors, notably in food processing. CP is capable of inactivating a broad spectrum of biological agents, including Gram-positive and Gram-negative bacteria, bacterial spores, viruses, prions, yeasts, and filamentous fungi. Its key advantage lies in the ability to achieve rapid and effective decontamination under ambient temperature and atmospheric pressure conditions, with minimal impact on food quality and relatively low operational costs, especially when ambient air is used as the working gas [97].

A device capable of generate plasma usually consists of two main components: (1) a high-voltage source providing energy through an electric field and (2) a reactor that applies this energy to a gas volume ionizing it into plasma [94].

**Figure 4:**
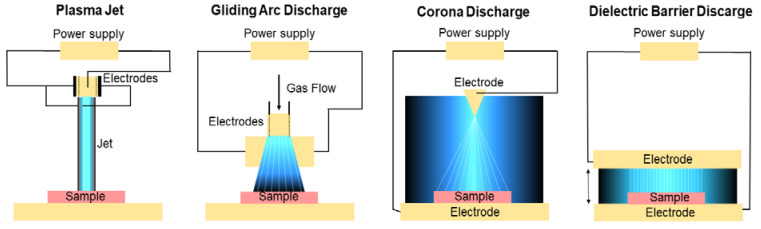
Schematic representation of different cold plasma (CP) generation systems, including plasma jet, gliding arc discharge, corona discharge, and dielectric barrier discharge (DBD). The plasma stream is illustrated in blue.

Various types of cold plasma systems have been developed, including dielectric barrier discharge (DBD), plasma jets, corona discharge, glow discharge, gliding arc plasma, or microwave discharge systems [97,98,99,100]. Figure 4 provides a schematic overview of several of these configurations. In all of these methods, the feed gas is typically energized into plasma. DBD and plasma jets are the most widely adopted due to their relatively simple design, adaptability, and scalability [101]. In the plasma state, free radicals, electrons, and ions are responsible for its conductivity, internal interaction, and significant responsiveness to electromagnetic fields.

A comprehensive exposition of the theoretical fundamentals of plasma chemistry is provided in Fridman’s seminal work [102]. The reactive species generated within the plasma are largely dependent on the type of gas used. When humid oxygen is employed as the plasma gas source, the generation of ROS is observed. The following are among the species present in such plasma: ^•^O, O(^3^p), ^•^OH, ^•^O_2_H, ^1^O_2_, ^•^O_2_^−^, O_2_^+^, O^+^, O^−^, O_3_, and H_2_O_2_ [103]. These species exhibit strong oxidative properties, particularly the hydroxyl radical (^•^OH), which is the neutral form of the hydroxide ion, and ozone (O₃), and they are primarily responsible for the effective inactivation of microorganisms.

In addition to UV radiation, the presence of molecular nitrogen (N₂) in the plasma leads to the generation of various reactive nitrogen species (RNS). Some of these species, such as ^•^N₂, N₂⁺, and N⁺, are short-lived and exist only within the plasma. Others, such as NO, N₂O, NO₂, and NO₃, are more stable and persist beyond the plasma region, where they can interact with oxygen to form additional reactive compounds. These nitrogen-containing molecules also contribute to the antimicrobial and biochemical activity of CP. The combined set of reactive oxygen and nitrogen species is commonly referred to as RONS [104].

When plasma is generated using inert gases such as helium (He), argon (Ar), or neon (Ne), the primary plasma constituents include their excited atoms, ions, and electrons. However, upon interaction with ambient air, the formation of RONS is similarly induced.

### 4.2. Mechanism of Action of Cold Plasma Technology (CPT) Against Fungi

Although numerous studies have sought to elucidate the mode of microbial inactivation by various types of plasma generated under atmospheric conditions, the precise mechanisms underlying microbial death are not yet fully understood [105]. Damage to the cell wall and the cytoplasmic membrane of microscopic organisms is primarily caused by reactive oxygen and nitrogen species (RONS) generated by cold plasma (CP). These reactive species induce oxidation and the breakage of chemical bonds in cell surface constituents. This process is followed by a leakage of intracellular constituents, such as proteins or nucleic acids [106,107]. Membrane rupture and desiccation allow cellular components to escape from the compromised cell, ultimately resulting in cell death. Furthermore, CP has been shown to reduce or impair energy production, thereby preventing spore formation and contributing to cell death [108].

The application of cold plasma generated by a gliding arc system to wheat grains contaminated with spores of *A. flavus* and *A. parasiticus* resulted in the degradation of spore DNA (particularly that of *A. flavus*), along with a significant reduction in aflatoxin levels, especially AFB_1_ and AFG_1_ [109]. CP has also been demonstrated to interfere with metabolic pathways and enzymatic processes essential for spore development [110]. In another study, atmospheric pressure DBD plasma was applied to *F. culmorum*. Following plasma treatment, disruption of the cell walls and membranes was observed, resulting in cytoplasmic efflux. These structural alterations became visible after 60 s of treatment, while cells exposed for 180 s exhibited a flattened morphology [111].

As reported by Misra et al. [97], fungal inactivation by CP may occur through several mechanisms, including the inhibition of cell membrane function during short exposure times, the induction of apoptosis, and intracellular nanostructural alterations. ROS oxidize intracellular organelles, particularly lipid phosphates, leading to the formation of lipid peroxides. The respective contributions of RNS and UV radiation remain underexplored due to the difficulty in isolating the individual effects of ROS and RNS. Therefore, further research is necessary to elucidate fungal responses specifically to UV and RNS derived from plasma sources. It has been proposed that fungal spores contain melanin in their cell wall layers, a pigment that confers resistance to external stressors, including UV radiation [112].

The antifungal mechanism of CP involves the generation of both ROS and RNS, which have been shown to destroy fungal cells, inhibit germination, and suppress mycotoxin biosynthesis [113]. Among these, the hydroxyl radical (^•^OH), a highly reactive and toxic species, can cleave covalent bonds in proteins, amino acids, phospholipids, DNA, and carbohydrates. It also induces lipid peroxidation and disrupts cell membranes. Another important species is the superoxide anion (^•^O₂^−^), which reacts with various biomolecules, including DNA, RNA, catecholamines, steroids, and lipids, contributing to lipid peroxidation. When fungal contamination is already present in food products, CP-generated RONS have been observed to degrade mycotoxins, either inactivating them or reducing their toxicity.

Importantly, CP treatment does not significantly alter the organoleptic properties or texture of food products, although some degree of lipid oxidation has been reported. To achieve optimal outcomes, the careful selection of operating conditions during plasma generation is required, as highlighted by de Oliveira et al. [113].

The interaction between CP and water has led to the development of plasma-activated water (PAW), an aqueous solution enriched with reactive and oxidizing species produced by plasma discharge. PAW possesses notable antimicrobial and oxidative properties, making it a versatile tool with applications in agriculture and food safety. Its efficacy has been demonstrated in areas such as crop protection, seed treatment, and plant growth enhancement [114].

### 4.3. Application of CPT to the Control of Toxigenic Fungi and Mycotoxins

CPT has been applied to control toxigenic fungi and reduce mycotoxin contamination in food. A growing body of the literature demonstrates that CP is an effective tool for degrading mycotoxins, including AFs, DON, enniatins, ZEA, and FUMs, as well as for inactivating mycotoxin-producing fungi, such as *Aspergillus*, *Alternaria*, *Penicillium*, and *Fusarium*, in a wide range of food products, including cereals and dairy commodities [95].

Spores of *A. parasiticus* exhibited a loss of integrity following 30 s of plasma exposure in a fluidized bed system, leading to the clustering of dispersed cell contents. These findings suggest that CP disrupts the cell wall, increasing its permeability and allowing the leakage of intracellular components [115].

In another study, roasted coffee samples inoculated with *A. westerdijckiae*, *A. steynii*, *A. versicolor*, and *A. niger* were incubated at 27 °C for 21 days to assess OTA production. A 6 min CP treatment completely inhibited fungal growth, while a 30 min treatment reduced OTA levels by 50%, indicating CP as a promising method for OTA reduction in food and feed production [116].

Atmospheric CP exposure of *A. ochraceus* spores resulted in their rupture and desiccation, significantly reducing spore viability [117]. DBD plasma was evaluated for decontaminating *A. niger*, *P. verrucosum*, *F. graminearum*, and *Rhizopus oryzae* on rice grains. Notably, a 4 min treatment reduced spore viability by 65–80% for *F. graminearum* and *R. oryzae* [118]. The mycelial growth of all tested fungi decreased substantially with increasing exposure time. The efficacy of CP is also influenced by spore size, with smaller spores more susceptible due to their higher surface-area-to-volume ratio.

CP has been demonstrated to degrade mycotoxins into less or non-toxic compounds in both solid and liquid matrices and in vitro. The degree of inactivation depends on various parameters, including the type of CP, applied voltage and frequency, exposure time, working gas composition, plasma–target distance, and contributions from UV radiation and reactive species [113]. CP significantly degrades AFs, with in vitro sensitivity following the order AFB1 > AFB2 > AFG1 > AFG2. AFs in contaminated hazelnuts were more resistant, though AFB₁ remained the most sensitive. After a 12 min exposure at 1150 W, residual AFB1 and total AF concentrations were 29.1% and 30.4%, respectively [119].

AFB1 and AFG1 contain a double bond at the 8,9-position of the furofuran ring, which, together with the cyclopentanone ring, contributes to their elevated toxicity and carcinogenicity. CP promotes the addition of H_2_O to this double bond, initiating chemical reactions that yield less toxic degradation products, as proposed by Zhao et al. [120]. The reaction of O_3_ with this double bond can also lead to epoxide formation and subsequent bond cleavage. Reduction percentages of AFB1 vary by matrix, from 45 to 56% in spiked rice and wheat food wheat to 95% on glass slides after 30 min of optimal corona discharge treatment [121]. Then, the AFB1 level reduction increases with the duration of CP sample exposure.

In corn powder treated with high-voltage atmospheric CP (HVACP), AFB_1_ reduction increased from 62% to 82% as exposure time extended from one to ten minutes [122]. Following the HVACP treatment of pure AFB_1_ on glass slides, six degradation compounds were identified, two of which were ozonolysis products, while the others indicated participation of additional reactive species [123].

In another study, AFs and OTA in polystyrene plate were treated with O_3_-based CP leading to an 81–82% reduction in AFB1 and AFG1 within 15 min and up to 99% after 60 min. In contrast, AFB_2_ and AFG_2_ were reduced by only 60%, and OTA by 70%. The toxicity of the resulting by-products remains unexplored. NOx-based CP demonstrated reduced efficacy: 64% and 63% reductions for AFB1 and AFG1, respectively, after 60 min, while OTA exhibited high resistance to this treatment. Similarly, in contaminated pistachio kernels treated with O_3_- or NO_x_-based CP, reduction percentages declined markedly, highlighting the matrix effect [124].

CP has also been effective against other mycotoxins, including FB_1_, ZEA, enniatin B, and sterigmatocystin (ST). In studies using pure standards of AAL toxin, DON, FB₁, ZEA, enniatin A, enniatin B, ST, and T-2 toxin, all were degraded within 60 s. However, in extracts from fungal cultures grown on rice (*F. verticillioides*, *F. avenaceum*, *F. graminearum*, *A. nidulans*), reduction rates were lower, particularly for FB_1_ and enniatin B, of which approximately 50% remained after treatment [125]. Suhem et al. [126] found that *A. flavus* mycelium appeared only after 20 days in CP-treated brown rice cereal bars stored at 25 °C and 100% humidity, following 20 min of 40 W exposure.

In groundnuts, CP exposure caused the complete disintegration of fungal spore membranes via electroporation and etching by reactive species. AFB1 levels were reduced by over 70% after 15 min at 40 W and over 90% after 12 min at 60 W [127]. CAP generated in ambient air achieved reductions of 93% for AFs, 90% for trichothecenes, 93% for FUMs, and 100% for ZEA after 8 min of exposure on glass coverslips [128]. Several additional studies confirm the potential of CP for both fungal inactivation and mycotoxin degradation [125,129,130,131]. The increasing adoption of this technology offers a promising approach to ensuring mycotoxin-free food on a global scale [132].

The expanding body of research indicates that CP will likely play a critical role in future strategies for controlling toxigenic fungi, eliminating mycotoxins, and targeting other microbial and chemical hazards, especially on food surfaces. CP treatments generally cause minimal changes to the physicochemical (pH, acidity, anthocyanins, respiration, vitamins) and sensory (color, taste, texture) properties of foods [133]. Food sectors that could benefit from the antifungal efficacy of CPT include fresh produce, food grains, nuts, spices, herbs, and dried meat and fish industries [97]. Nonetheless, further research is needed to define optimal CP conditions tailored to each food type without compromising organoleptic quality.

In the United States, the use of CP in food and food packaging has been approved by the Food and Drug Administration (FDA), Environmental Protection Agency (EPA), and the U.S. Department of Agriculture (USDA). In the European Union, the EFSA oversees the assessment of new food technologies, and only food products receiving EFSA marketing approval are permitted [133].

## 5. Lactic Acid Bacteria (LAB)

Lactic acid bacteria (LAB) are a group of non-spore-forming, Gram-positive bacteria that use carbohydrates as their sole or primary carbon source [134]. They primarily produce acid lactic through the fermentation of carbohydrates. LAB are generally cocci or rod-shaped and exhibit high tolerance to low pH, high salt concentrations, and heat treatments. They are anaerobic or facultative anaerobes and are found in diverse environments. They have been used since ancient times in various domains of human activity.

Certain LAB are classified as GRAS by the US FDA, and some of them hold a “qualified presumption of safety” (QPS) status by the EFSA (EFSA Panel on Biological Hazards) [135]. These LAB strains are known for their ability to reduce microbial and mycotoxin contamination in food and may serve as valuable biocontrol agents.

**Figure 5:**
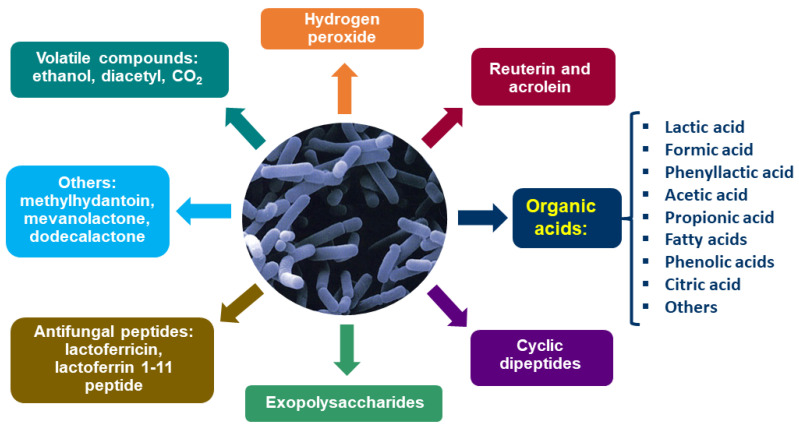
Some antifungal compounds produced by lactic acid bacteria.

The antifungal activity of selected LAB strains against toxigenic fungi can be attributed to both competitive growth and the production of a wide range of antifungal metabolites. These include organic acids such as lactic, phenyl lactic, propionic, benzoic and acetic acids, as well as CO_2_, ethanol, H_2_O_2_, diacetyl, proteinaceous compounds, low-molecular-weight compounds (e.g., reuterin, cyclic dipeptides, hydroxylated fatty acids, methylhydantoin, and mevalonolactone), and bacteriocin-like substances [136,137,138,139]. Figure 5 illustrates some of the key compounds produced by LAB.

In 2020, Zheng et al. [140] proposed the reclassification of the genus *Lactobacillus* based on biochemical, phylogenetic, and taxonomic differences among its constituent groups and species. As a result, the original genus was split into 25 genera, including the emended genus *Lactobacillus, Paralactobacillus* and twenty-three newly established genera. The novel genera include the following: *Amylolactobacillus*, *Agrilactobacillus*, *Acetilactobacillus Apilactobacillus*, *Bombilactobacillus*, *Companilactobacillus*, *Dellaglioa*, *Fructilactobacillus*, *Furfurilactobacillus*, *Holzapfelia*, *Lacticaseibacillus*, *Lactiplantibacillus*, *Lapidilactobacillus*, *Latilactobacillus*, *Lentilactobacillus*, *Levilactobacillus*, *Ligilactobacillus*, *Limosilactobacillus*, *Liquorilactobacillus*, *Loigolactobacilus*, *Paucilactobacillus*, *Schleiferilactobacillus*, and *Secundilactobacillus.*

In the literature, the nomenclature of LAB used against fungi prior to the publication by Zheng et al. [140] is still predominant (e.g., *Lactobacillus casei*, *L. pentosus*, *L. acidophilus*, *L. delbrueckii*, *L. sakei*, *L. brevis*, *L. rossiae*, *L. reuteri*, *L. helveticus*, *L. fermentum*, *L. paraplantarum*, etc.) [137]. LAB employed to prevent fungal contamination in food include *Lactiplantibacillus plantarum* (formerly *Lactobacillus plantarum*) [140], *L. salivarius* [141], *Pediococcus pentosaceus*, *Leuconostoc mesenteroides*, *Lacticaseibacillus paracasei*, *Latilactobacillus sakei*, *Companilactobacillus farciminis*, and *Levilactobacillus brevis*, among others [142,143].

### 5.1. Mechanisms of Action of LAB on Fungi and Mycotoxins

LAB possess the capacity to reduce mycotoxin levels in contaminated food through a variety of mechanisms. LAB can act against toxigenic fungi by inhibiting or, more commonly, retarding their growth. Since mycotoxins are typically produced during the late stages of fungal development, the inhibition or retardation of fungal growth can result in the absence of mycotoxin production in the medium. This inhibition may be due to competition for space and nutrients, the production of organic acids, antifungal metabolites, or a combination of these factors [144]. Although LAB produce a range of metabolites (Figure 5), organic acids, primarily lactic, phenyllactic, propionic, and acetic acids, appear to play a major role in antifungal activity. Nevertheless, other metabolites are also considered important contributors [137].

In addition, LAB can reduce the accumulation of mycotoxins in food matrices through several mechanisms, including chemical or enzymatic degradation, metabolic conversion and adsorption. In the latter process, mycotoxins bind to components of the LAB cell wall, thereby preventing their absorption into the food/feed [145]. The ability of LAB to bind mycotoxins is largely due to the composition of their cell wall, which contains peptidoglycans, linear glycan chains consisting of N-acetylglucosamine and N-acetylmuramic acid cross-linked by short peptides. Variations in the amino acid sequences of these peptides can lead to structural differences in the peptidoglycans among LAB strains [146]. In addition to peptidoglycans, teichoic acids have been shown to be involved in the binding of AFB1 by *L. casei* Shirota and *Lactobacillus reuteri* [147]. Other cell wall components such as proteins and β-D-glucans also contribute to AFB1 binding through hydrogen bonding and van der Waals interactions [137].

Physicochemical changes in the environment, such as variations in pH or temperature, may affect the binding capacity of LAB to AFB1, making this interaction reversible. These environmental factors influence the surface properties and electrostatic interactions involved in AFB1 binding. Among the mechanisms reported in the literature, the cell wall binding of AFB1 by LAB is the most commonly described.

In the case of OTA, binding was shown to be influenced by cell surface hydrophobicity, as well as electron donor–acceptor and Lewis acid–base interactions [148]. The influence of ethanol (5%) on the OTA-reducing ability of *Oenococcus oeni* in a wine-like medium initially containing 2 µg of OTA/L has been demonstrated in both live and heat-inactivated cells. This effect was attributed to an adsorption-based mechanism [149,150]. Notably, mycotoxin adsorption occurs in both live and heat-inactivated LAB.

For the removal of type-A trichothecenes, adsorption to the LAB cell wall has been identified as the principal mechanism [151,152,153]. Approximately 51% of the type-B trichothecene deoxynivalenol (DON) was eliminated from sourdough bread through binding to the cell wall of *L. plantarum* [154]. Similarly, a strain of *L. paracasei* eliminated 41% of DON through an adsorption mechanism involving cell wall components. This strain bound 39% of DON, and nearly 33% was removed within 20 h in a phosphate-buffered solution [155].

Regarding FUMs, the adsorption of FB1 and FB2 by *L. paraplantarum* and *Streptococcus thermophilus* was investigated by Niderkorn et al. [146], who suggested that peptidoglycan or structurally related compounds are likely responsible for FUM binding. Although the precise mechanism of interaction between FUM and LAB cell wall peptidoglycan remains unclear, it has been proposed that the tricarboxylic acid chains of FUMs interact with peptidoglycans during the binding process.

ZEA can interact with LAB strains through three potential mechanisms: (a) electrostatic or hydrophobic interactions with surface proteins, (b) adsorption to peptidoglycan, or (c) penetration into the cell, where it may interact with intracellular proteins [156].

Another mechanism of action by which LAB mitigate mycotoxins is degradation, wherein bacterial metabolites chemically alter the mycotoxin structure, yielding other compounds. For instance, ZEA can be reduced to α-zearalenol (ZOL) [146,157]; OTA may be degraded into ochratoxin α and L-β-phenylalanine, likely via peptidases from *Pediococcus parvulus* [158]; and PAT can be transformed into hydroascladiol through two intermediates (E- and Z-ascladiol) by *L. plantarum* [159]. Despite these capabilities, adsorption is generally the preferred mechanism, as it does not lead to the formation of harmful degradation products in food matrices.

### 5.2. Application of LAB Against Toxigenic Fungi and Mycotoxins

Numerous studies have reported the effectiveness of LAB in inhibiting toxigenic fungi and reducing mycotoxins, primarily in in vitro assays using appropriate solid culture media. LAB are commonly grown on de Man, Rogosa and Sharpe (MRS) agar, which is then overlaid with media conducive to fungal development, such as Czapek yeast agar, 20% sucrose, or cereal-based media [142,143]. Other suitable media for fungal growth have also been employed. Certain LAB strains have demonstrated biocontrol potential against toxigenic fungi belonging to the genera *Aspergillus*, *Fusarium*, and *Penicillium* in food systems [160].

MRS broth has been used to obtain cell-free supernatant (CFS) by the centrifugation of LAB cultures, enabling the study of metabolites produced in contaminated matrices. Nazareth et al. [161] reported that the CFS of *Lactiplantibacillus plantarum* grown in MRS broth inhibited the growth of *A. flavus* and *F. verticillioides* on contaminated corn kernels and ears, respectively. The application of this extract led to a reduction in mycotoxin production in corn kernels and ears ranging from 73.7% to 99.7% compared to controls.

However, some fungi such as *A. flavus*, *Penicillium roqueforti*, *Cladosporium* spp., and *A. niger* exhibited high resistance to antifungal metabolites produced by *L. plantarum*, with 60% to 80% of the strains failing to delay their growth. Conversely, 75% of *L. plantarum* strains showed strong inhibitory activity against *F. culmorum*, *P. expansum*, and *P. chrysogenum* [162]. Certain *L. plantarum* strains also demonstrated the ability to extend the shelf life of bread.

In one study, 1400 LAB isolates were screened in vitro for antifungal activity against *A. niger*, using inhibition zone measurements to assess activity. *L. plantarum* strain YML007 exhibited the highest antifungal activity, also showing inhibitory effects against *A. flavus*, *A. oryzae*, and *F. oxysporum*. Analysis of the CFS indicated that a novel protein was responsible for the antifungal activity, although its mechanism of action remains unknown [163].

Juodeikiene et al. [164] investigated the impact of LAB on DON, ZEA, and T-2/HT-2 toxin levels in malting wheat grains contaminated with *Fusarium* spp., particularly *F. culmorum*. The application of *Lactobacillus sakei* KTU05-6, *Pediococcus acidilactici* KTU05-7, and three strains of *Pediococcus pentosaceus* significantly reduced ZEA, DON, and T-2/HT-2 levels, with reductions ranging from 23% to 73%. These reductions were attributed to either the binding or detoxification of mycotoxins by the LAB.

In another study, CFS from 16 LAB strains cultured in MRS broth under anaerobic conditions and obtained via centrifugation was tested against *A. parasiticus* and *P. expansum* using the disk diffusion method. LAB strains showing antifungal activity were then used in bread fermentation alongside yeast to examine their effects on fungal growth and AF reduction in bread previously inoculated with *A. parasiticus*. Six LAB strains (*Bifidobacterium bifidum*, *Lactobacillus ruminis*, *L. rhamnosus* (CECT 288), *L. johnsonii*, *L. plantarum*, and *L. bulgaricus*) at a 20:1 concentration ratio were effective in inhibiting *P. expansum* growth. When incorporated into bread manufacturing with yeast, these strains reduced AF levels in contaminated bread samples by 84.1% to 99.9%. The addition of *L. plantarum* and *L. bulgaricus* also extended shelf life by 3 to 4 days [165].

The strains *L. plantarum* Q1-2 and *L. salivarius* Q27-2 were employed as inoculants in mixed fermenting feed. Compared to the control, these strains reduced AFB1 levels by 34.17% and 16.57% and DON levels by up to 90.61% and 51.03%, respectively [141].

Eleven selected LAB strains were also tested for their ability to reduce or inhibit the growth of *A. flavus*, *A. parasiticus*, *A. carbonarius*, *A. niger*, *A. welwitschiae*, *A. steynii*, *A. westerdijkiae*, and *P. verrucosum*, and to reduce AF and OTA production. Dual culture antifungal assays were performed using MRS agar overlaid with Czapek yeast 20% sucrose agar at various temperatures. *P. verrucosum* and *A. steynii* were the most sensitive to LAB treatment, while *A. niger* and *A. welwitschiae* were the least. Two *Pediococcus pentosaceus* strains exhibited the most significant antifungal activity. Reductions in AFB1 levels ranged from 19% to 55% in *A. parasiticus* and 22.8% to 52.3% in *A. flavus*. AFB2 concentrations were reduced by 20.7% to 60.8% in *A. parasiticus* and by 16.2% to 57% in *A. flavus*. OTA reductions ranged from 7.5% in *A. niger* to complete elimination (100%) in *P. verrucosum* [142].

In a separate study using dual cultures in MRS-cereal-based medium, the same LAB strains demonstrated the ability to inhibit the growth of various *Fusarium* species and reduce mycotoxin production [143]. The observed order of fungal susceptibility to the tested LAB strains was as follows: *F. oxysporum* > *F. poae* = *F. culmorum* ≥ *F. sporotrichioides* > *F. langsethiae* > *F. graminearum* > *F. subglutinans* > *F. verticillioides*. Overall, the most effective LAB strain was *Leuconostoc mesenteroides* ssp. *mesenteroides* (T3Y6b), while the least effective strains were *Latilactobacillus sakei* ssp. *carnosus* (T3MM1 and T3Y2). The efficacy of LAB strains in reducing mycotoxin levels varied depending on the specific LAB strain, the type of mycotoxin, and the ambient temperature.

Byrne et al. [166] evaluated the effect of six LAB isolates on the development of Fusarium head blight (FHB) under both in vitro and glasshouse conditions. The study assessed the relative expression of *Fusarium* marker genes and transcription factors in response to infection. In dual-culture assays, inhibition zones covered up to 10% and 17% of the total plate area for *Limosilactobacillus amylovorus* FST 2.11 and *Levilactobacillus brevis* R21, respectively. The antifungal activity of these isolates was further confirmed through detached leaf assays, which showed the significant suppression of sporulation by *F. culmorum* and *F. graminearum*. Additionally, mycotoxin analysis revealed that *L. amylovorus* DSM20552 significantly reduced DON content in spikelets.

Eight *Lactiplantibacillus pentosus* strains were assessed for their ability to remove ZEA from a sodium acetate buffer solution, with initial ZEA concentrations ranging from 5.51 to 74.70 μg/mL. The results indicated a positive correlation between ZEA concentration and adsorption capacity. Strain JM0812 showed the highest adsorption rate, reaching 83.17% in the solution with 74.70 μg/mL of ZEA, followed by strains UM054 (82.78%) and UM055 (81.69%) [167].

Metabolites produced by LAB have also been employed as food preservatives, aiming to protect products from fungal spoilage and extend shelf life. Lactic acid, indolelactic acid, and 4-hydroxyphenyllactic acid, produced by *L. plantarum* and *Lentilactobacillus buchneri*, have been shown to inhibit the growth of *P. nordicum* and suppress mycotoxin synthesis [168]. Additionally, phenyllactic acid has been observed to inhibit the growth of mycotoxigenic strains such as *P. verrucosum* and *P. citrinum* [169].

Synthetic antifungal preservatives used in food products, including benzoates, sorbates, and propionates, have raised health concerns, ranging from irritability and inattentiveness to carcinogenic effects and neurological damage. LAB have emerged as safer and more desirable alternatives for antifungal preservation [170]. Consequently, LAB strains are expected to play a significant role in future applications, not only as probiotics but also as natural preservatives.

## 6. Nanoparticles

### 6.1. Concept

The definition of nanoparticles (NPs) remains a topic of ongoing discussion. According to the International Organization for Standardization (ISO), a nanoparticle is defined as a nano-object with all three external dimensions in the nanoscale, typically ranging from 1 to 100 nanometers. When these dimensions differ significantly, alternative terms such as “nanofiber” or “nanoplate” are more appropriate [171]. Previously, the Scientific Committee on Consumer Products (SCCP) [172] defined nanoparticles as particles with diameters between 1 and 100 nm in at least one dimension. However, a universally accepted definition for “nanomaterial” and “nanoparticle” has yet to be established, complicating regulatory development [173].

### 6.2. Classification of Nanoparticles

Nanoparticles can be classified into lipid-based, polymeric, carbon-based, and inorganic categories, which can be further subdivided based on their composition [174]. Lipid-based NPs typically manifest as spherical platforms comprising at least one lipid bilayer surrounding one or more internal aqueous compartments and are frequently utilized as delivery systems. Examples include liposomes, lipid nanoparticles, and oil/water emulsions [175,176].

Among inorganic NPs, there are silica NPs (crystalline or amorphous), metal NPs (MNPs) such as copper, titanium, aluminum, iron, nickel, lead, selenium, cobalt, cadmium, gold, and silver [177], metal oxides like titanium dioxide, iron oxide, copper oxide, zinc oxide, and magnesium oxide [178], as well as bimetallic NPs or quantum dots composed of semiconducting materials like silicon. The advancement of nanotechnology across various fields has led to an increase in the variety and complexity of NPs.

This review primarily focuses on metal or metal oxide NPs, which have been utilized in the treatment of toxigenic fungi. Silver nanoparticles (AgNPs) have garnered particular attention in recent years due to their antibacterial, antiviral, and antifungal activities.

### 6.3. Nanoparticle Properties

The biological properties of NPs are primarily attributed to their high specific surface area, resulting from their small size and large surface-to-volume ratio. Additional factors influencing their behavior include shape, pore size, surface charge, surface atom density, and whether the structure is crystalline or amorphous.

While NPs exhibit a wide range of properties (optical, electrical, catalytic, antibacterial, and medical) this review focuses on their antifungal activities.

Among the most commonly employed NPs for controlling fungal growth are zinc oxide nanoparticles (ZnO-NPs) and silver nanoparticles (AgNPs), with copper nanoparticles (CuNPs) also utilized [179].

ZnO-NPs are noted for their superior durability, selectivity, and thermal stability compared to other antimicrobial agents. Research into ZnO as an antimicrobial began in the early 1950s [180], but its application gained momentum in 1995 when its efficacy against various bacterial strains was demonstrated. Although bulk ZnO exhibits antimicrobial properties, ZnO-NPs demonstrate significantly enhanced efficiency against pathogens due to their reduced size [181]. ZnO-NPs have shown notable antifungal activity against a variety of fungi, including *A. flavus* and *A. fumigatus* [182], *A. niger*, *F. oxysporum,* and *P. funiculosum* [183], the toxigenic phytopathogen *F. graminearum*, the postharvest fungus *P. expansum* [180], *Alternaria citri* [184], and phytopathogenic fungi such as *Botrytis* spp., *Penicillium* spp., and *Pilidiella granati* [185].

Among metal nanoparticles, AgNPs are stable, exhibit electrical conductivity, and have long been employed as antimicrobial agents, able to destroy bacteria, fungi, and viruses at very low concentrations, and are highly effective against fungal pathogens.

### 6.4. Mechanisms of Action Against Fungi and Mycotoxins

The antifungal activity of metal NPs (MNPs) is primarily attributed to electrostatic interactions between metal ions and microbial cell membranes, leading to membrane damage and the disruption of intracellular organelles [181]. However, there is a variety of targets in the cell. Among the MNPs, AgNPs have been observed to operate through multiple mechanisms, including binding to phosphate groups in DNA and interactions with the plasma membrane that cause proton diffusion and subsequent cell death [186].

The mechanisms by which NPs affect pathogenic fungi are less thoroughly studied compared to bacteria [187]. Fungal cells possess a complex structure comprising a cell membrane surrounded by a dynamic cell wall. The cell wall is primarily composed of the polysaccharide glucan, situated between an external layer of mannoprotein and an internal layer of the polysaccharide chitin, and is subject to constant remodeling in response to proliferation and environmental challenges. The cell wall contains membrane-bound glucan synthases. The cell membrane contains transmembrane proteins and ergosterol units, in addition to the phospholipid bilayer [43].

AgNPs have demonstrated the ability to inhibit fungal growth and induce morphological and metabolic changes [188,189]. For instance, in *F. graminearum*, AgNP treatment has been associated with the production of ROS after six hours, leading to increased malondialdehyde levels and elevated protective enzyme activity [190]. Additionally, the application of 45 ppm AgNPs resulted in a significant decrease in the production of oxalic, maleic, and citric acids, as well as a reduction in mycotoxin production (up to 80%) and alterations in the enzymatic profile in *A. niger* and *P. chrysogenum* [188]. However, further research is necessary to elucidate the interaction of NPs with individual components of fungal cells [1].

In *Ustilaginoidea virens*, AgNPs have been reported to inhibit growth by disrupting cell wall and membrane integrity, interfering with intracellular structures, and altering genome-wide transcription. Interestingly, an upregulation of mycotoxin ustilaginoidin biosynthetic genes was also observed [187].

**Figure 6:**
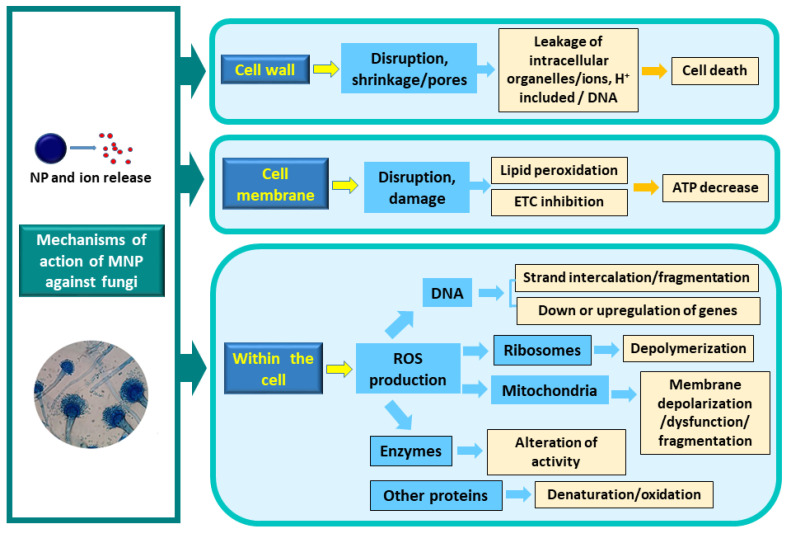
Possible mechanisms of action of metal nanoparticles in fungal cells.

A schematic representation of the potential mechanisms through which MNPs exert their effects in fungal systems is illustrated in Figure 6. While specific mechanisms of action on each fungal cellular target depend on the particular MNP and are not yet fully understood, general observations include the release of ions both extracellularly and intracellularly, which bind to specific protein groups. This binding can affect the function of critical membrane proteins and disrupt cell permeability. MNPs have been shown to inhibit conidial germination and suppress development. Furthermore, MNPs and their released ions disrupt electron transport (ETC) chains, promote protein oxidation, and alter membrane potential. The release of ROS in significant quantities is a hallmark of these processes leading to mitochondrial membrane depolarization and increased transcription of certain genes. Ribosomes may undergo depolymerization, and ROS can trigger oxidation reactions catalyzed by different MNPs, causing severe damage to proteins (including enzymes), membranes, DNA, and interfering with nutrient absorption. The genotoxic effects of ions and ROS on DNA ultimately lead to cell death. In the case of AgNPs or CuNPs, the released ions can bind to sulfhydryl groups of proteins, including enzymes, leading to denaturation [43,181,186].

### 6.5. Application of Nanoparticles Against Toxigenic Fungi and Mycotoxins

To date, research has predominantly focused on antibacterial NPs, with comparatively limited studies addressing antifungal NPs and their effects on toxigenic fungi and mycotoxin production in food. Applications targeting phytopathogenic fungi generally follow two primary strategies: (1) encapsulating antifungal compounds within polymeric nanocages, and (2) utilizing NPs alone to exert inhibitory effects. The second approach primarily employs MNPs due to their stability, rapid action, and potential for green synthesis [1].

The synthesis route of MNPs can significantly influence their antifungal activity, as residual metal precursors or surfactants may be challenging to remove. These by-products can alter the surface chemistry of the NPs, thereby affecting their antifungal efficacy [191].

Asghar et al. [192] evaluated the efficacy of Fe, Cu, and Ag NPs against *A. flavus* and *A. parasiticus*, observing antifungal activity in the following order: AgNPs > CuNPs > FeNPs. AgNPs demonstrated potent antifungal activity against these fungal strains and inhibited AF production.

Tarazona et al. [193] reported that chemically synthesized AgNPs measuring 30 nm exhibited remarkable in vitro activity against various toxigenic *Fusarium* species, including *F. graminearum*, *F. culmorum*, *F. sporotrichioides*, *F. langsethiae*, *F. poae*, *F. oxysporum*, *F. proliferatum*, and *F. verticillioides*. The impact of these AgNPs on the control of the main aflatoxigenic *Aspergillus* species (*A. flavus*, *A. parasiticus*) and ochratoxigenic species (*A. carbonarius*, *A. niger*, *A. ochraceus*, *A. steynii*, *A. westerdijkiae*, and *Penicillium verrucosum*) affecting foods was also studied [194]. *A. flavus* and *A. parasiticus* exhibited reduced sensitivity to AgNPs compared to the ochratoxigenic fungi evaluated. The study found that fungal growth and mycotoxin accumulation in cultures generally decreased with increasing AgNP doses and longer exposure time of spores to AgNPs.

The activity of AgNPs with diameters of 2, 15, and 60 nm against fungicide-sensitive and fungicide-resistant *F. graminearum* strains was studied in vitro [195]. The study revealed a correlation between AgNP activity and particle size, with 2 nm AgNPs exhibiting the highest activity. However, despite the antifungal properties of AgNPs, an increase in DON production was observed.

Biosynthesized AgNPs have also demonstrated the capacity to impede the growth of *A. niger*, *P. chrysogenum*, *F. culmorum*, and *Alternaria alternata* [196]. Ammar and El-Desouky [197] reported that biosynthesized AgNPs at a concentration of 220 μg/100 mL of media reduced OTA production in *A. ochraceus* by 52–59%.

Reports on the utilization of MNP types other than AgNPs against toxigenic fungi are limited. Among these, zinc oxide (ZnO) nanoparticles are the most studied [198,199]. ZnO-NPs have been shown to reduce the ability of *P. expansum* and *F. oxysporum* to produce fusaric acid and patulin, respectively, in a concentration-dependent manner [198]. The supplementation of ZnO-NPs and/or probiotics has been demonstrated to ameliorate the toxic changes resulting from DON toxicity, suggesting that combining ZnO-NPs with probiotics in poultry feed can reduce the need for high ZnO-NP doses, thereby mitigating potential toxicity concerns [199]. ZnO and Fe_2_O_3_ NPs were assayed in vitro against *A. flavus* and found to be effective in reducing fungal growth, hence avoiding AF production [200]. ZnO@SiO_2_ nanocomposites, comprising ZnO nanoparticles as the core and mesoporous silica encapsulation, were evaluated against *A. flavus* and *F. graminearum* in maize flour medium and sterile kernels. The results demonstrated the substantial inhibition of fungal hyphae growth and morphological disruption, with antifungal effects exhibiting a dose-dependent response. Concurrently, a decline in mycotoxin production (AFs, DON, and ZEA) was observed [201].

Considering the impact of NPs on mycotoxin production by toxigenic species belonging to the genera *Aspergillus*, *Fusarium*, *Penicillium*, and *Alternaria*, it is evident that the absence of fungal growth often correlates with reduced or absent mycotoxin production. The AgNPs utilized in these studies are predominantly spherical, with diameters typically exceeding 10 nm, possibly to minimize toxicity to humans and animals.

The efficacy of NPs against both fungicide-sensitive and fungicide-resistant fungi is optimized when particle sizes are below 5 nm; however, low sizes results in increased toxicity to non-target cells, although the shape is also relevant to toxicity [202]. It is important to note that most research on the antifungal activity of NPs has been conducted in vitro. In some cases, in vivo applications have been performed on crops at a small scale. The direct application of NPs to pathogens is preferable, considering the differences between controlled laboratory environments and the complexities of field conditions.

The number of publications addressing the use of NPs in food, either directly or as components of food containers/packaging to prevent or delay the growth of toxigenic fungi and mycotoxin production, remains limited. Incorporating AgNPs into food packaging materials has the potential to effectively delay food deterioration; however, concerns exist regarding NP migration into the packaged food. To address these concerns, further research is necessary to determine methods that allow for the safe use of NPs, ensuring protection for both consumers and the environment from potential contamination.

A promising yet scarcely explored approach involves the direct action of NPs to reduce or inhibit the toxicity of mycotoxins through adsorption or degradation. The potential of carbon-based nanomaterials, including fullerenes, graphenes, magnetic graphenes, and nanodiamonds, as well as hybrid nanoparticles and inorganic montmorillonite nanocomposites, to bind mycotoxins through adsorption has been demonstrated [1]. Consequently, the use of NPs for managing toxigenic fungi and mycotoxins emerges as a promising strategy that warrants further investigation to achieve the complete inhibition of these species, thereby safeguarding humans, animals, and the environment from their hazardous effects.

### 6.6. Biosafety Issues in Food Applications

While AgNPs exhibit promising antifungal and anti-toxigenic activity in food systems, their inclusion in food products or packaging raises significant biosafety concerns that must be carefully considered before widespread adoption. The toxicological effect of AgNPs have been reviewed [202,203,204,205,206,207,208]. However, the biotransformation and bioaccumulation of AgNPs inside the organisms is not well understood. The toxicity of AgNPs is extensively studied using different model systems (e.g., algae, bacteria, yeast, water flea, brine shrimp, fish, embryonic and adult zebrafish, mice, rat, and plants). These investigations have concluded that there are different mechanisms of toxicity of NPs, and their toxicity and behaviors depend on factors such as size (lower sizes imply more toxicity), shape (spherical NPs are less toxic than rods or plates), structure, agglomeration state, surface charge, wettability, dose, and substance type [202,206,207]. The routes of entry of NPs in humans are ingestion, inhalation, dermal contact, and directly in systemic circulation via injection [209,210]. Oral exposure to AgNPs, either directly through treated food or indirectly via migration from food-contact materials, may act on the mucus layer, be absorbed, and translocated to the blood stream through the gastrointestinal tract. Within enterocytes, AgNPs can trigger oxidative stress, DNA damage, and inflammation [210]. Once internalized, AgNPs can interact with gastrointestinal fluids, potentially releasing ionic silver (Ag⁺), which is biologically active and more toxic. They can accumulate in systemic organs such as the liver, kidneys, and spleen, causing oxidative stress, inflammation, and genotoxicity [211]. They may disrupt cellular functions, mitochondrial activity, and DNA integrity, posing risks such as cytotoxicity and immunotoxicity [211]. AgNPs exhibit low-to-moderate bioavailability, though size, coating type, and aggregation state greatly influence absorption and distribution. Ingested AgNPs may disrupt the balance of gut microbiota, a key determinant of human health. Specific concerns include the following: reduction in beneficial commensal bacteria, induction of dysbiosis, which could have downstream effects on metabolism, immunity, and gut barrier integrity or potential increase in antimicrobial resistance genes within the microbiome due to prolonged sublethal Ag exposure. They may also traverse the blood–brain barrier and affect the nervous system [208]. The EFSA evaluated the use of AgNPs as additives in plastic packaging material for food. They concluded that, while these nanoparticles remain embedded in non-polar plastics and do not migrate significantly, there is a release of Ag^+^ up to 6 μg/kg of food, which is below the established value of 50 μg/kg of food and the acceptable daily intake (ADI) of 0.9 μg/kg body weight per day. However, EFSA noted that exposure to silver from other dietary sources could exceed the ADI, raising potential safety concerns [212].

## 7. Magnetic Materials

### 7.1. Concept

An emerging methodology for mitigating mycotoxins in food involves the use of magnetic materials. These materials consist of small particles with a magnetic core, typically at the nanoscale, coated with a non-magnetic layer. This coating facilitates the binding of target molecules through adsorption or degradation reactions and serves to protect the magnetic core from oxidation or migration. Functionalization of the coating with diverse chemical groups has been shown to enhance the versatility of these particles [213]. Following interaction with the target compounds, the magnetic particles can be efficiently removed from the substrate using an external magnetic field. These materials encompass both nanostructured and micrometer-sized particles, representing an advancement over conventional inorganic adsorbents such as bentonites or zeolites commonly employed for mycotoxin adsorption in food products.

### 7.2. Synthesis

The synthesis of magnetic nanomaterials (MNMs) commonly utilizes magnetic materials, including iron (magnetite and maghemite), cobalt, manganese, nickel, and their derivatives. Iron oxides, particularly Fe_2_O_3_ and Fe_3_O_4_, along with ferrite derivatives like CoFe_2_O_4_ and MnFe_2_O_4_, are predominantly used in the production of MNMs due to their relative simplicity of preparation, chemical stability, high magnetic moments, and compatibility with biological systems, which makes them suitable for a wide range of applications [213].

Magnetic nanostructured agents are typically synthesized via wet chemical procedures with specific methods varying based on the desired structure. For instance, magnetite nanoparticles (Fe_3_O_4_-NPs) can be synthesized by the reverse coprecipitation method, which involves the gradual addition of FeCl_3_ and FeSO_4_ solutions to an ammonium hydroxide solution at 60 °C under ultrasonic agitation. The resulting Fe_3_O_4_-NPs are then magnetically separated, thoroughly washed with distilled water, and prepared for subsequent applications [214].

The preparation of magnetic alginate beads involves incorporating Ca^2^⁺ as a cross-linking agent into a Fe_3_O_4_-NPs suspension, followed by the addition of sodium alginate. The formed beads are then magnetically separated, washed, and dried. These magnetic alginate porous beads are particularly attractive due to their high specific surface area, rapid recovery, cost-effectiveness, and chemical versatility, making them suitable for combination with other materials to enhance contaminant affinity [214].

The addition of non-magnetic materials to the magnetic suspension is also feasible. For example, the fabrication of spherical magnetic alginate-activated carbon beads involves adding sodium alginate and activated carbon to a Fe_3_O_4_-NPs suspension containing Ca^2^⁺ [215,216].

Other compounds utilized in the preparation of these magnetic agents include aluminum hydroxide nanoparticles, bentonite, esterified pectin, chitosan, nanocellulose, and ordered mesoporous carbon [217]. The materials bound to the magnetic core can also serve to immobilize enzymes capable of degrading harmful compounds such as mycotoxins.

According to Targuma et al. [213], the magnetic core of these nanoparticles may comprise various materials, including maghemite (γ-Fe_2_O_3_), magnetite (Fe_3_O_4_), neodymium, and magnetic alloy nanomaterials such as iron-nickel (FeNi), iron-cobalt (FeCo), iron-platinum (FePt), or iron-palladium (FePd) alloys.

### 7.3. Application of Magnetic Materials to Mycotoxin Detoxification

Magnetic nanomaterials (MNMs) have been primarily applied to remove mycotoxins from liquids such as water, fruit juices, milk, wine, and other beverages, rather than for controlling fungal growth.

A study investigated the effectiveness of a magnetic graphene oxide (MGO) nanocomposite, synthesized from iron oxide nanostructures and graphene oxide (GO), as an adsorbent for reducing *Fusarium* mycotoxins in naturally contaminated palm kernel cake, which is used as animal feed. The targeted mycotoxins included ZEA, FB1, FB2, DON, HT-2, and T-2 toxins. Optimal reduction was achieved at pH 6.2, temperature 40.6 °C, and a contact time of 5.2 h, resulting in reduction levels of 69.57% for DON, 67.28% for ZEA, 57.40% for HT-2, and 37.17% for T-2 [218].

Zhang et al. [219] synthesized a novel magnetic reduced graphene oxide composite (Fe_3_O_4_@rGO) using a one-step solvothermal method, successfully applied it to AFB1 adsorption. The study provides new insight into the preparation of magnetic composite adsorbents and presents a potential candidate for use in the food industry.

A nanocomposite made of the fungal mycelium of *A. flavus*, graphene oxide, and Fe_3_O_4_ and named FM@GO@Fe_3_O_4_ was successfully prepared by a two-step process including co-culturing and the hydrothermal method. This material presented characteristics of mesoporous structure and superparamagnetic behaviors exhibiting excellent adsorption performances for AFB1 and ZEA [220].

The efficacy of magnetic zeolite nanocomposite (MZNC) as an adsorbent for reducing mycotoxins in barley flour has been reported. The nanocomposite demonstrated high effectiveness, removing over 99% of AFs, 50% of OTA, 22% of ZEA, and 1.8% of DON from contaminated samples. Additionally, adsorption by MZNC was found to be superior to that of natural zeolite [221].

González-Jartín et al. [222] examined the efficiency of 25 types of magnetic nanocomposites for binding mycotoxins from contaminated aqueous solutions. Nanocomposites with diameters less than 15 µm, composed of mixtures of activated carbon, bentonite, and aluminum oxide, were capable of eliminating up to 87% of mycotoxins from solutions containing DON at 12.5 µg/L, ZEA at 50 µg/L, FB1 at 50 µg/L, and AFB1, AFB2, AFG1, and AFG2 at 1.75 µg/L. The nanocomposites demonstrated an adsorption efficiency of 450 µg/g. Spheres measuring less than 3 mm and composed of biopolymers and activated carbon or GO have been shown to remove up to 70% of mycotoxins through adsorption (598 ng/g). The mycotoxins are adsorbed to the coating surrounding the magnetic material. After a designated time, the application of a neodymium-iron-boron (NdFeB) magnet permits their separation from the solution. This method was applied to contaminated beer. The most effective particles were found to be those composed of mixtures of alginate and activated carbon or pectin, which were able to completely eliminate AF and OTA from the contaminated beer and significantly reduce the levels of FB1 and ZEA in the sample. The authors concluded that the use of magnetic nanostructured particles during brewing can contribute to reducing mycotoxin content and identified magnetic NMs as promising adsorbents for mycotoxin removal from beverages.

PAT adsorption by chitosan-coated Fe_3_O_4_ nanoparticles reached a maximum capacity of 6.67 mg/g within 5 h when 300 μg of adsorbents were added to 10 mL of an aqueous solution containing 200 μg/L of PAT. Additionally, the recovery rate of the chitosan-coated Fe_3_O_4_ reached 99.95% within 60 min, demonstrating excellent recoverability. Moreover, toxicological experiments indicated that chitosan-coated Fe_3_O_4_ particles posed minimal safety concerns, suggesting their potential application in PAT adsorption in future studies [223]. However, potential impact on sensory attributes may occur.

Chitosan-coated magnetic particles (Fe_3_O_4_) have demonstrated effectiveness in adsorbing PAT from fruit juice. However, interactions between the coated magnetic particles and other components in the juice may occur, potentially impacting sensory attributes such as taste, aroma, color, or texture. PAT adsorption by chitosan-coated Fe_3_O_4_-NP reached a maximum capacity of 6.67 mg/g within 5 h when 300 μg of adsorbents were added to 10 mL of an aqueous solution containing 200 μg/L of PAT. Additionally, the recovery rate of the chitosan-coated Fe_3_O_4_ reached 99.95% within 60 min, demonstrating excellent recoverability. Moreover, toxicological experiments showed that chitosan-coated Fe_3_O_4_ particles posed safety concerns, suggesting their potential application in PAT adsorption in future studies [223].

Maize waste-derived magnetic carbon nanocomposites were used to remove AFB1 in poultry birds fed with contaminated feed, achieving an adsorption ratio of nearly 90% within 3 h at pH 7.0. The findings indicate that the prepared adsorbent can serve as an alternative to powdered activated carbon for the detoxification of AF in poultry feed, as activated carbon can lead to dehydration and salt deficiencies when administered to poultry. The adsorbent was found to be nearly non-toxic to poultry, and its removal from feces was straightforward using a magnetic apparatus [224].

A novel PAT-degrading enzyme preparation was developed by covalently linking a short-chain dehydrogenase/reductase (CgSDR) using genipin as a cross-linking agent to dopamine/polyethyleneimine co-deposited magnetic Fe_3_O_4_ particles. The enzyme had previously been shown to degrade PAT. Optimal immobilization provided 63% immobilization efficiency and 62% activity recovery. When these magnetic particles were introduced into juices contaminated with PAT, the mycotoxin was converted into non-toxic E-ascladiol by the enzymatic reaction. The immobilized enzyme exhibited combined effects of PAT adsorption by Fe_3_O_4_ particles and biological degradation by CgSDR, achieving a PAT detoxification rate of 100% in phosphate-buffered saline and over 80% in apple juice. The immobilization protocol substantially improved thermal and storage stabilities, proteolysis resistance, and reusability [225]. The mechanism of action of this magnetic material involves biochemical degradation of the mycotoxin PAT rather than adsorption.

The application of magnetic materials to remove mycotoxins from bulk solid food matrices has not been reported. However, MNMs have been utilized in analytical separation procedures (clean-up) of mycotoxins from matrices prior to their detection and quantification by chromatographic techniques. This technique is referred to as magnetic solid-phase extraction [226,227]. These materials have been incorporated into a novel extraction procedure, namely, dispersive magnetic solid-phase extraction, which has been successfully applied to extract AF in paprika [228].

## 8. Irradiation

### 8.1. Concept

Radiation refers to the emission or transmission of energy in the form of waves or particles through space or a material medium.

### 8.2. Classification

Radiation may be classified into two categories:

(a) Ionizing radiation, which possesses sufficient energy to remove tightly bound electrons from atoms, thereby ionizing them. Examples include X-rays, gamma rays (γ-rays), and electron beams. Gamma rays are typically produced by radioisotopes such as cobalt-60 (^60^Co) or cesium-137 (^137^Cs). X-rays can have energies up to 5 MeV, while electron beams can reach energies up to 10 MeV [229].
(b) Non-ionizing radiation, which lacks the energy required to ionize atoms or molecules. This category includes UV radiation, visible light, infrared radiation, microwaves, and radio frequencies. Notably, UV radiation lies at the boundary between ionizing and non-ionizing radiation; while most UV radiation is non-ionizing, the highest-energy UV rays can exhibit ionizing properties.

### 8.3. Usage of Irradiation in Food

Irradiation is a well-established method for eliminating potential contaminants such as insects, parasites, bacteria, viruses, and fungi in food products. This technique has been employed since the mid-20th century. Beyond microbial decontamination, irradiation offers additional benefits, including the inhibition of sprouting and the extension of shelf life [230]. Its widespread and approved use for sterilizing and decontaminating various food types results in minimal impact on the safety and organoleptic properties of the products.

In 2005, the quantities (in tons) of irradiated food were as follows: 183,309 in Asia and Oceania, 116,400 in America, 90,035 in Africa, Ukraine, and Israel, and 15,060 in Europe. The primary categories of irradiated food included spices and dried vegetables (45%), garlic and potatoes (22%), grains and fruits (20%), meat and seafood (8%), and others (4%) [230].

The energy absorbed during irradiation is typically expressed in grays (Gy) or kilograys (kGy), where one gray equals one joule per kilogram of product. The mechanism of food irradiation involves disrupting the genetic material (DNA and RNA) of microorganisms, thereby preventing their reproduction or pathogenicity. This can occur directly or indirectly, as irradiated water molecules generate free radicals and ROS that damage genetic material. Additionally, irradiation targets enzymes and proteins that contribute to spoilage, thus enhancing the shelf life of food [231].

In the food industry, three irradiation levels are commonly recognized:

(a) Low dose (Radurization): 0.1–1 kGy, used to inhibit respiration, delay ripening, disinfect pests, and inactivate parasites such as *Trichinella*.
(b) Medium dose (Radicidation): 1–10 kGy, applied to reduce spoilage and eliminate microbial pathogens like *Salmonella* spp. This level is utilized for frozen foods and is analogous to pasteurization, albeit without thermal energy.
(c) High dose (Radappertization): >10 kGy, typically employed for sterilization, effectively destroying all microorganisms in food products, including spores [231].

Despite its efficacy against microbial and parasitic organisms, irradiation can influence the amino acid composition of food proteins, potentially affecting their digestibility. It may also alter vitamin C content and induce lipid oxidation, leading to changes in organoleptic properties.

### 8.4. Effect of Irradiation on Toxigenic Fungi and Mycotoxins in Food

According to Dadachova and Casadevall [232], fungi appear to interact with ionizing radiation in a manner distinct from other terrestrial organisms. In particular, fungi, especially melanized species, exhibit a high degree of resistance to radiation when exposed to elevated doses under experimental conditions. Melanin, a naturally occurring pigment found in skin and certain fungi, possesses significant antioxidant properties. It has the ability to absorb and scatter radiation, thereby shielding cells from direct damage. This protective mechanism is particularly effective in neutralizing free radicals generated by radiation, which may otherwise lead to DNA damage and compromise other cellular structures. Additionally, melanin has been associated with enhanced fungal proliferation.

The overall impact of ionizing radiation on fungal growth can be attributed to its capacity to disrupt physiological processes and cellular integrity. Molecular mechanisms involved include DNA fragmentation and mechanical damage to cell walls, caused by destabilization of the lipid bilayer and membrane-associated proteins [233]. The effectiveness of irradiation is influenced by several factors, including the administered dose, the physiological state of the fungi, and their morphological characteristics. In the context of food safety, irradiation not only inhibits fungal proliferation but also contributes to the degradation of certain mycotoxins [234,235,236].

As reported by He and Zhou [237], the irradiation of feedstuffs induces a spectrum of physical, chemical, and biological effects, which collectively contribute to the reduction or elimination of mycotoxins.

Khalil et al. [238] investigated the effects of varying doses of gamma irradiation on the growth of *A. flavus* and *A. ochraceus* in artificially inoculated yellow maize. The study also assessed the impact of irradiation on the production of AFB1 and OTA. A dose of 6.0 kGy completely inhibited fungal growth, while a dose of 4.5 kGy significantly reduced mycotoxin production. The most pronounced degradation of AFB1, AFB2, and OTA occurred at 20 kGy, with reduction rates of 40.1%, 33.3%, and 61.1%, respectively.

In another study, copra (dried *Cocos nucifera* L.) contaminated with *A. flavus* spores was subjected to various gamma irradiation doses (0.3, 0.7, 1.0, and 3.0 kGy) [239]. A dose-dependent decline in fungal counts was observed, with 0.7 kGy proving especially effective, achieving a significant reduction in fungal load in compliance with regulatory limits. The most substantial decrease in fungal contamination was recorded at 3.0 kGy, confirming the efficacy of gamma irradiation against *A. flavus*.

Wheat grains inoculated with *A. flavus* and pretreated with calcium oxide (CaO) were exposed to gamma radiation (0–20 kGy). Within the maximum permitted dose of 10 kGy for cereals, a combined treatment of 0.5% CaO and 10 kGy was proposed, achieving a fungal reduction rate between 42% and 89%, along with the mitigation of AFB1 and AFB2 contamination [240].

Rice seeds contaminated with fungi, including *Aspergillus* spp. and *Alternaria* spp., were exposed to gamma irradiation at doses ranging from 1 to 6 kGy. A marked reduction in fungal populations was observed at 1–2 kGy, with complete eradication achieved at doses exceeding 4 kGy. While *Aspergillus* spp. did not survive at 4 kGy, *Alternaria* spp. persisted. These differences highlight the varying radiosensitivities of fungal genera. Notably, seed germination potential remained largely unaffected, although alterations in membrane permeability were observed, which may impact the nutritional integrity of seeds [241].

The effectiveness of irradiation against fungal pathogens such as *Alternaria* spp. and *Fusarium* spp. has also been evaluated. *Alternaria* spp., characterized by melanized cell walls, demonstrate notable radioresistance. The presence of melanin plays a critical role in preserving viable spores during irradiation. It has been hypothesized that melanin-containing fungi can convert gamma radiation into metabolic energy, potentially enhancing growth under sublethal radiation doses. This hypothesis is supported by the findings of Dadachova and Casadevall [232], who demonstrated that radiation energy can be transformed into chemical energy for fungal growth.

According to Liu et al. [242], AFB1 content in various cereals can be reduced by 43.0–87.8%, 65.7–71.5%, 22.0–100%, and 90.7% when treated with gamma rays, electron beams, UV light, and microwave radiation, respectively. DON can be decomposed by 37.0–82.4% (gamma rays), 17.2–56.3% (electron beams), and 83.4–100% (UV light) in feedstuffs. ZEA can be decontaminated by 25.0–86.0% (gamma rays) and 60.0–100% (UV light) in grains. FB1 was inactivated by 63.5–100% (gamma rays), 58.1% (electron beams), and 93.3% (microwave radiation) in feedstuffs.

UV irradiation has also demonstrated high efficacy in mycotoxin decontamination. Treatment with UV light at a wavelength of 365 nm achieved reductions exceeding 90% in AF content from various nuts [243]. Furthermore, this wavelength effectively eliminated AFB1 in peanut oil.

Fruits and vegetables inoculated with fungi responded positively to irradiation treatments, with higher doses leading to more pronounced fungal inhibition in oranges [244]. However, product quality must be considered, as irradiation may alter the color and texture of fresh produce. Additionally, the sensory properties of irradiated food products such as *bahulu* cakes and bread were found to be affected by the irradiation dose [245]. Bandyopadhyay et al. [246] reported that gamma irradiation at 5 kGy reduced firmness in food, while a lower dose of 2 kGy ensured microbial safety in vegetable toppings and fillers without significantly affecting sensory attributes such as texture and flavor.

The variable efficacy of irradiation in mycotoxin degradation is influenced by treatment conditions, including dosage, exposure time, and the morphological and compositional characteristics of the feedstuffs [243,247]. While irradiation presents a promising method for mycotoxin decontamination, safety concerns remain, particularly regarding potential mutagenic effects and the emergence of harmful microorganisms, as well as the potential degradation of nutritional quality. Further research is warranted to address these challenges and facilitate the safe and effective application of irradiation technologies [242].

### 8.5. Regulations

The safety of irradiated foods for human consumption has been the subject of scrutiny due to concerns regarding the potential of ionizing radiation to induce chemical changes. A substantial body of research, both national and international, has been devoted to evaluating the health implications of irradiated foods. This body of work has been systematically reviewed and assessed by joint expert committees of the International Atomic Energy Agency (IAEA), the World Health Organization (WHO), and the Food and Agriculture Organization (FAO) of the United Nations. These expert panels have concluded that food irradiation does not pose any increased toxicological, microbiological, or nutritional risk beyond those associated with conventional food processing techniques [248].

In the United States, the FDA has conducted a comprehensive evaluation of the safety of irradiated foods and has determined the process to be safe. This position has also been endorsed by the Centers for Disease Control and Prevention (CDC) and the United States Department of Agriculture (USDA).

Within the EU, the regulatory framework governing the use of irradiation in the processing of food and food ingredients is established under Directive 1999/2/EC [249], which remains in force and has not been replaced. The sale of irradiated food and food ingredients in the EU market is permitted only when such items are included in the list of approved food categories as specified in Directive 1999/3/EC [250]. Currently, the only categories authorized for irradiation and commercial distribution within the EU are dried aromatic herbs, spices, and vegetable seasonings.

## 9. Machine Learning Methods

### 9.1. Key Concepts

The term artificial intelligence (AI) refers to a machine’s ability to perform operations that typically require human intelligence, such as speech recognition, natural language understanding, and decision making [251]. AI has become increasingly pervasive in modern society, manifesting in various forms including news media, social media platforms, and the scientific literature.

The field of AI is experiencing exponential growth due to its vast applications across numerous disciplines. It is evolving rapidly within computer science and has demonstrated notable advancements in medical and biological research. The principal objective of AI is to develop systems capable of emulating human cognitive abilities, thereby enabling them to perform complex tasks and solve real-world problems. AI encompasses several subfields, including machine learning (ML), deep learning (DL), natural language processing, computer vision, robotics, and data mining [252], all of which work synergistically to replicate aspects of human intelligence.

Machine learning (ML), a prominent subfield of AI, enables computer programs to learn from data and to identify complex patterns and associations that may be indiscernible to the human brain [253]. ML is widely applied to analyze large datasets and uncover patterns that relate to both known and previously unknown outcomes.

ML is inherently multidisciplinary, drawing from probability theory, statistics, and approximation theory. It comprises a range of techniques, including supervised, unsupervised, semi-supervised, and reinforcement learning.

In supervised learning, the dataset consists of both input and output data. The input data are labeled with known outputs corresponding to the research objective. This approach utilizes training sets derived from known categories to develop predictive models. The process begins with the preliminary categorization of each data element in the training set by a human operator. The ultimate goal of supervised ML is to construct a model that optimally correlates input variables with the desired output. The effectiveness of this approach is evidenced by its wide application in regression and classification tasks, enabling the model to make accurate predictions when presented with previously unseen data.

Unsupervised learning methods, by contrast, aim to identify patterns and inherent structures in input data without relying on predefined output labels. In this paradigm, input data are provided without corresponding correct outputs [254], allowing the model to group data into clusters based on similarities, a process known as clustering.

Semi-supervised learning combines elements of both supervised and unsupervised learning, leveraging a limited amount of labeled data alongside a larger volume of unlabeled data to improve clustering accuracy.

Reinforcement learning involves algorithms that interact with the environment to learn how to perform a specific task by selecting an optimal sequence of actions that maximize cumulative rewards over time [255,256].

**Figure 7:**
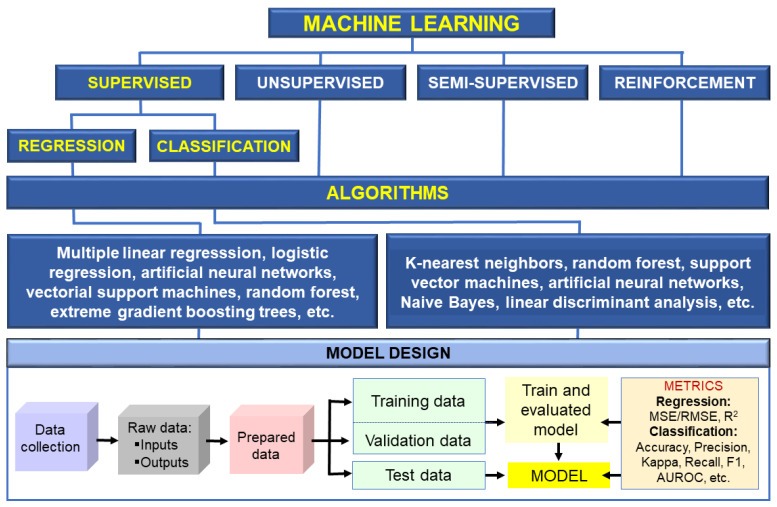
Machine learning overview and model design workflow, with a focus on supervised learning approaches. Some algorithms are shared between regression and classification.

To perform these tasks, ML methods employ a variety of algorithms (structured sets of instructions that guide the system in performing calculations or solving problems) (Figure 7). The suitability of a particular algorithm for a given task, such as regression or classification, plays a crucial role in determining its effectiveness and practical application.

In the domain of mycotoxicology, supervised ML models have emerged as a highly effective approach for controlling toxigenic fungi and mycotoxins. These models, designed for regression or classification tasks, have demonstrated remarkable efficacy. The present study focuses on elucidating the mechanisms and applications of these models in the control of toxigenic fungi and mycotoxins.

Developing a supervised ML model involves a series of essential steps:

(a) Data collection, which should be as extensive and reliable as possible, encompassing both input (features) and output (outcome) raw data;
(b) Data preparation or preprocessing, which typically includes cleaning and formatting the raw data into a usable form. Categorical variables must be encoded numerically to be processed by computers;
(c) Splitting of the preprocessed dataset into three subsets: training data, validation data, and test data. These are typically divided in proportions of 50%, 25%, and 25%, respectively. However, these ratios may vary, and, in some cases, the training and validation sets are combined through a method called cross-validation;
(d) Selection of models and algorithms to be tested depending on the particular task (e.g., multiple linear regression (MLR), multilayer neural networks (MLP-NNs), random forest (RF), support vector machines (SVMs), decision trees (DTs), extreme boosting gradient trees (XGBoost), k-nearest neighbors, etc.);
(e) Selection of the programming environment (e.g., R, Python or Julia, along with their corresponding packages, caret, scikit-learn, or MLJ);
(f) Training the model using the training data and evaluating it using the validation data or through the k-fold cross-validation process. During this phase, the hyperparameters of the algorithms are tuned to optimize model performance. For regression tasks, this involves minimizing the error between predicted and actual numerical values, while, for classification tasks, the objective is to maximize classification performance based on metrics such as accuracy, precision, recall, or F1 score;
(g) Final model evaluation using the test set, which is not involved in the training or validation phases [254] (Figure 7).

Model performance is evaluated using appropriate statistical metrics. For regression models, common metrics include the mean squared error (MSE), the root mean squared error (RMSE), and the coefficient of determination (R^2^). When comparing multiple regression models, the optimal choice is typically the one with the lowest MSE or RMSE and the highest R^2^ value (approaching or equal to 1).

For classification models, a wide array of metrics is used to assess performance. These include the confusion matrix, accuracy, precision, recall (or sensitivity), the receiver operating characteristic (ROC) curve, area under the ROC curve (AUROC), F1 score, Cohen’s kappa, log loss (or cross-entropy loss), and others [257,258].

A potential challenge in building ML models is overfitting, which arises when the model performs very well on training data but fails to generalize effectively to new, unseen data. In this context, regularization serves as a critical technique, introducing a penalty for model complexity to prevent overfitting. In situations where large datasets are unavailable, k-fold cross-validation is a widely adopted method to ensure robustness.

**Figure 8:**
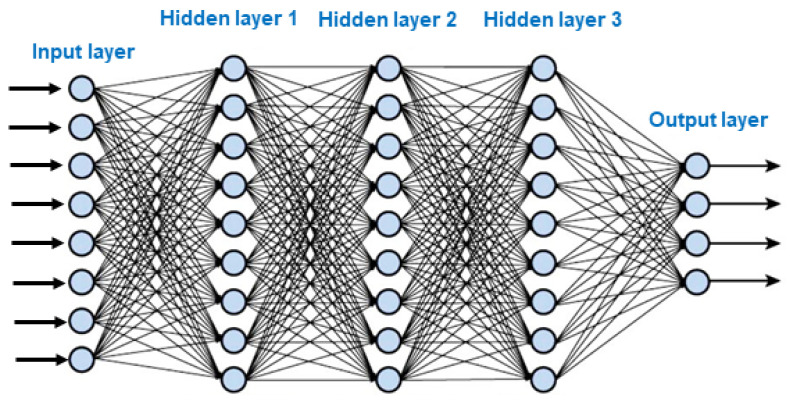
Topology of a deep neural network comprising an input layer, three hidden layers, and an output layer. Each layer consists of interconnected nodes (neurons), illustrating the hierarchical structure used for feature extraction and decision-making.

Deep learning (DL), a rapidly growing subfield of ML, employs artificial neural networks (ANNs) with multiple hidden processing layers. DL can incorporate supervised, unsupervised, or reinforcement learning approaches. Figure 8 presents a schematic of a deep neural network with three hidden layers of nodes (neurons).

Convolutional neural networks (CNNs) are a type of artificial neural network (ANN) characterized by complex architectures. These networks have proven effective in handling tasks that involve large datasets, such as analyzing millions of data points, each containing numerous features. This is particularly useful when the features exhibit distinct structural relationships, such as adjacent pixels in an image [259,260]. CNNs have become a widely used technology in modern applications due to their ability to automatically identify key features without the need for manual input, as demonstrated in numerous studies [261]. This capability is especially evident in tasks like hyperspectral image analysis.

DL has shown particular success in tasks involving large-scale (thousands of data points) and high-dimensional datasets. However, it is important to note that DL demands significantly more computational resources compared to traditional ML techniques. Additionally, the “black box” nature of DL models, with their opaque processing layers, presents challenges related to model interpretability and accountability [262].

**Figure 9:**
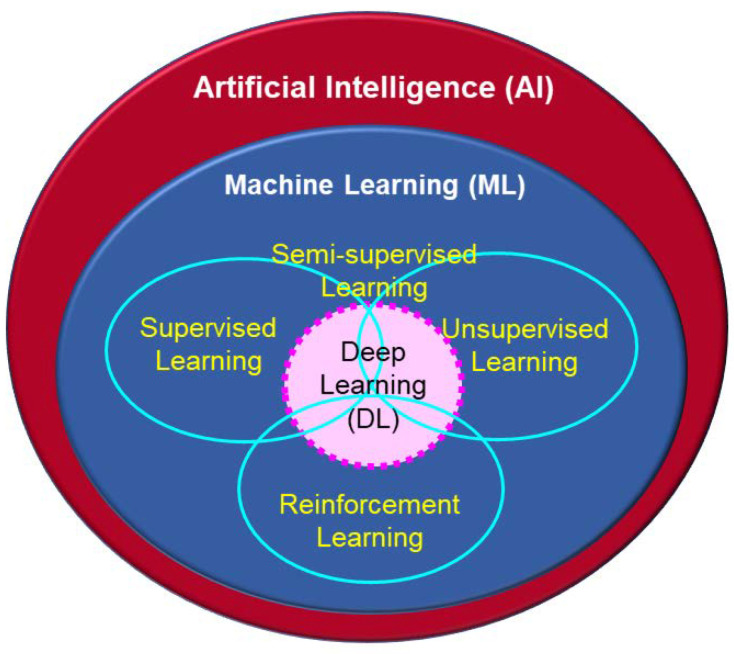
Relationship between artificial intelligence (AI), machine learning (ML), and deep learning (DL).

Figure 9 presents a schematic illustrating the relationships between AI, ML, and DL. It is worth underlining that some algorithms are shared between supervised regression and supervised classification. The use of ANNs is evident across supervised regression, supervised classification, and DL applications.

### 9.2. ML and DL Applications in Modeling Growth/Identification of Toxigenic Fungi and Mycotoxin Production

The application of AI in mycology has witnessed significant growth in recent years, with notable advancements in the identification of fungi and the detection of mycotoxin contamination in food commodities. The integration of AI with a variety of analytical platforms, including spectroscopy, biosensors, and imaging techniques, holds great potential. This integration is expected to enhance the efficiency, reliability, and speed of mycotoxin analysis [261]. One example is computer vision, which simulates the human visual system and is considered a promising detection technology due to its advantages of high detection speeds, low cost, ease of maintenance, and high visualization.

In this context, *Aspergillus nidulans*, *A. niger*, *A. oryzae*, *A. versicolor*, and *P. citrinum* were cultured on unhulled paddy samples [263], and images of the developed colonies were captured using an optical system (Sony Nex-6 digital camera with a Sony SELP1650 lens). Various algorithms were employed to construct models for fungal identification, including SVMs, backpropagation neural networks (BP-NNs), CNNs, and deep belief networks (DBNs). The model built using the DBN achieved 100% accuracy in identifying the fungi present in the samples based on the images.

To monitor AFB1 levels in edible oils, input data were obtained through Raman spectroscopy using a DXR laser Raman spectrometer (Thermo Fisher Scientific, Waltham, MA, USA). CNNs and recurrent neural networks (RNNs) were used to develop models capable of identifying and monitoring AFB1 levels in the samples. The RNN model demonstrated superior detection performance compared to the CNN model in detecting AFB1 contamination levels in edible oils [264].

An olfactory visualization approach was employed to detect AFB1 in peanuts. A colorimetric sensor was specifically designed to assemble an olfactory visualization system, which was subsequently used to capture odor characteristic data from a series of peanut samples. A genetic algorithm (GA) combined with a BPNN regressor was then applied to optimize the color component of the preprocessed sensor feature image [265]. Following this, a Support Vector Regression (SVR) model was constructed using the optimized combination of characteristic color components to quantify AFB1 in peanuts. Two optimization approaches were compared: the grid search (GS) algorithm and the sparrow search algorithm (SSA). The results revealed that the SSA-SVR model, utilizing a combination of seven characteristic color components, yielded the most accurate predictions.

An electronic nose (e-nose) array (AIR PEN 3 (Airsense Analytics GmbH, Schwerin, Germany) equipped with a 10-metal oxide sensor array, was employed for the rapid identification of mycotoxins (AFB1 and fumonisins (FUMs)) in maize [266]. To determine whether the e-nose could differentiate between samples with concentrations above or below legal limits for AFB1 or FUMs, the study utilized ANN, logistic regression, and linear discriminant analysis (LDA). All methodologies demonstrated high accuracy (≥70%) in distinguishing maize contamination levels relative to the legal limits, with the ANN model outperforming the others, achieving 78% accuracy for AFB1 and 77% for FUM.

The levels of DON in different genetic lines of barley kernels were assessed using hyperspectral imaging (HSI) and CNNs. The hyperspectral system was a PlantSpec 10 (Middleton Spectral Vision, Middleton, WI, USA). Classification models were constructed using various algorithms, including linear regression (LR), SVM, CNN, RF, k-nearest neighbors (KNN), and stochastic gradient descent (SGD) [267]. The results indicated that CNNs showed significant potential in discriminating DON levels in the samples, achieving an accuracy of 89.8%.

The use of HSI to evaluate damage caused by Fusarium head blight (FHB) in wheat kernels following the inoculation of experimental fields with *F. graminearum* has been investigated by Dhakal et al. [268]. At harvest, wheat kernels were analyzed for DON content using gas chromatography–mass spectrometry (GC-MS). The well plate containing the kernels was scanned using a benchtop HSI system Pika L 2.4 (Resonon Inc., Bozeman, MT, USA). The effectiveness of HSI in disease classification (symptomatic vs. non-symptomatic) and its correlation with DON content and kernel damage was assessed. Nine ML methods were evaluated to determine the most accurate approach for classifying varying degrees of kernel damage. These included Gaussian Naïve Bayes (NB), (K-NN), LDA, (MLP NN), RF, SVM with linear and radial basis function kernels, gradient boosting (G-Boost), and partial least squares discriminant analysis (PLS LDA). The model developed using G-Boost yielded the best results, achieving 97% accuracy in classifying wheat kernels according to severity level. Furthermore, a deep neural network method, Mask R-CNN, was employed to segment individual kernels, classify them based on the proportion of symptomatic pixels, count the number of kernels in a sample, and correlate these parameters with DON content.

In another study, Han and Gao [269] applied a CNN as the primary DL approach to detect AFs in peanuts. Data inputs were collected using HSI, which provide continuous spectral information corresponding to an infinite number of color channels. The system contains a Grating spectrometer module (GSM, V8E, Dualix Spectral Imaging, China, 292–865 nm about 1 nm spectral bandwidth), a SCMOS CCD (ORCA-Flash2.8 C11440-10C, HAMAMATSU, Japan), an electric displacement platform (HG-32-TA, Beijing uh guano Dakota Co., Ltd., China) and a UV 365 nm LED illumination source (HRC-UV-4A, Shenzhen Weihai Lixin Technology Development Co. Ltd., China). The CNN approach demonstrated superior accuracy compared to traditional recognition models, achieving recognition rates exceeding 96% at the pixel level and 90% at the kernel level, highlighting its effectiveness in detecting AF contamination in peanuts.

A comprehensive evaluation of several classical DL algorithms was conducted to detect *A. flavus* contamination and determine contamination timing in peanut kernels using HSI acquired using a short-wave infrared hyperspectral imaging system (Isuzu Optics Corp., Taiwan, China). A total of 240 kernels per peanut group were imaged daily from day 1 to day 7. Six algorithms were analyzed, among which the dual-aspect attention spatial-spectral transformer (DAASST) algorithm demonstrated the highest accuracy, with overall classification accuracies of 99.4% and 100% for eight (seven contaminated plus one control) and two (contaminated vs. control) peanut classes, respectively [270]. Notably, LDA, a classical ML algorithm with a simpler architecture, also yielded high performance, with classification accuracies of 92.71% and 100% for the same peanut class groups, respectively.

Kim et al. [271] developed a compact convolutional transformer (CCT)-based model to classify wheat contaminated by DON and AFs into three categories: healthy, incipient, and contaminated. Classification was based on elevated CO_2_ respiration rates and the visual appearance of fungal development in RGB images captured using a digital camera (Galaxy S23+, Samsung Electronics, Rep. Korea) at both early and advanced stages of storage. The proposed CCT model achieved an overall accuracy of 83.33%, with the contaminated class showing the highest performance metrics: precision (1.0), recall (0.90), and F1 score (0.95), followed by the healthy and incipient classes.

Several studies have also focused on developing ML models to predict the growth of toxigenic fungi and/or mycotoxin accumulation under controlled environmental in vitro conditions. Mateo et al. [272] reported the use of ML models to predict OTA accumulation by *A. carbonarius* in grape juice-based solid media. These models utilized temperature, water activity (a_w_), time, and carbendazim dose as inputs and were developed using either MLP NNs or radial-basis function networks (RBFNs). Additionally, these algorithms have been applied to model DON accumulation in barley seeds inoculated with *F. culmorum* under different environmental conditions, including varying temperatures, a_w_ levels, inoculum sizes, and incubation periods [273]. RBFNs tended to produce lower errors and demonstrated better generalization performance compared to MLP NNs, although they required a larger number of hidden nodes.

Recent research has employed various ML algorithms, including MLP NN, RF, XGBoost) and multiple linear regression (MLR), to develop models predicting the growth rate (GR) of *F. culmorum* and *F. proliferatum* as well as the accumulation of ZEA and FB1 and FB2 in maize extract medium. These predictions were made under the influence of different commercial antifungal treatments, temperatures, and a_w_. Among the tested models, XGBoost produced the most accurate predictions for both fungal growth and mycotoxin production, yielding the lowest RMSE and the highest R^2^ values [274].

The application and comparison of various algorithms were carried out to develop predictive models for fungal growth and toxin production by *F. culmorum* and *F. proliferatum* in solid cultures treated with ethylene vinyl alcohol (EVOH) copolymer films containing pure components of EOs, under varying a_w_ and temperature conditions [275]. In this study, the RF algorithm produced the most accurate predictive model, whereas the MLR algorithm demonstrated the poorest performance. The same algorithms, along with SVMs, were used to construct ML models for accurately predicting the GR of *F. sporotrichioides* and the production of T-2 and HT-2 toxins by this species in autoclaved oat kernels exposed to EVOH films containing EO components [59]. The XGBoost algorithm yielded the highest predictive performance, despite its relatively high computational complexity. Following XGBoost, the best-performing models were RF and MLPNN, while SVM ranked fourth, and MLR again exhibited the lowest performance.

In a separate study, ML models were developed to predict the growth inhibition of various toxigenic fungi in vitro in the presence of different LAB strains, and their performance was compared. The most effective ML models for predicting the growth of different *Aspergillus* spp. and *P. verrucosum* were obtained using the MLP NN, followed by RF [142]. In contrast, when the experiments were conducted with toxigenic *Fusarium* spp., XGBoost and RF produced highly accurate models for predicting the percentage of fungal growth inhibition [143].

The transition from controlled laboratory environments to field conditions, where crop growth is influenced by meteorological variables and agricultural practices, often necessitates the inclusion of additional contextual data. In this context, Torelli et al. [276] employed NNs to predict contamination levels of maize with FUM, DON, and ZEA in Northern Italy. The input layer consisted of 15 nodes representing agronomic and meteorological variables. Agronomic variables included geographic location, seed FAO class, sowing and harvest times, the presence or absence of irrigation, pesticide application, and grain moisture at harvest. Meteorological variables included minimum and maximum temperatures and precipitation levels. The model achieved an R^2^ value of 0.57 for the regression line comparing predicted and actual values in the FUM test dataset.

The use of unmanned aerial vehicles (UAVs) to monitor wheat diseases has gained traction due to their capacity for high spatial resolution and flexible data acquisition timing [277]. The researchers utilized in the experiment an UAV M600 Pro aircraft of Daijang Innovations (DJI), and a Cubert S185 FireflEYE SE hyperspectral imaging camera. Hyperspectral UAV images spanning wavelengths from 450 to 950 nanometers were used to monitor FHB, a disease associated with DON contamination, in wheat fields. These images were used as input for an enhanced BP-NN model to assess its performance in classifying wheat plots as either slightly contaminated with FHB (<30%) or severely contaminated (>30%). Several ML algorithms were evaluated for this classification task. The BP-NN model achieved the highest overall accuracy (98%), followed by Fisher’s LDA), RF, and the SVM, each with 95% accuracy. The results demonstrated that hyperspectral imaging via UAVs can be effectively leveraged for FHB monitoring in winter wheat.

Another study conducted by Wang et al. [278] explored the use of ML algorithms to predict the probability of the presence of one or more mycotoxins in wheat across European regions. Specifically, the study investigated the use of satellite imagery to predict DON contamination in wheat crops in the Netherlands. Satellite images covering the sampling locations used in the field survey in the Netherlands were derived from Landsat 8 (L8) surface reflectance product (USGS Landsat 8 Collection 1 Tier 1 and Real-Time data OLI Raw Scenes). The RF algorithm, using a combination of weather data and wheat phenology as input, delivered high predictive performance. The model categorized contamination risk into three levels: low, medium, and high. When satellite imagery alone was used as input, predictive accuracy was moderate; however, combining satellite imagery with weather and phenology data substantially improved model performance, achieving an overall prediction accuracy of 0.81. These findings underscore the enhanced predictive potential of integrated data sources for regional-scale risk assessments of mycotoxin contamination in cereal crops.

Two deep neural network (DNN) models were trained to predict, at harvest, whether maize fields were contaminated with AFB1 and fumonisins (FB1 + FB2) above the legal limits. The input dataset included variables collected across multiple years, comprising five categorical variables (maize hybrid FAO class, previous crop, the week of sowing, the week of harvest, and European corn borer damage) and several continuous variables (growing days, days from sowing to harvest, and a calculated variable for kernel moisture at harvest). Both DNN models achieved an accuracy exceeding 75%, highlighting the added value of ML approaches over classical statistical methods in predictive modeling for mycotoxin contamination [279].

Soil properties, in combination with weather data and historical AF levels, were utilized to develop gradient boosting machine (GBM) models for identifying AF-contaminated maize in Iowa [280]. The GBM model demonstrated an overall accuracy of 96.77% for AF prediction with a balanced accuracy of 50% at a 20 ppb risk threshold. For a 5 ppb threshold, the model maintained a high overall accuracy (90.32%) and improved balanced accuracy (64.88%). According to the authors, such predictive models are both practical and feasible for implementation in commodity grain handling systems, enabling a shift from reactive to preventive mycotoxin mitigation strategies.

Similarly, in Illinois, historical data on AF and FUM contamination in maize, along with daily weather records, satellite imagery, dynamic geospatial soil properties, and land use data, were employed to identify significant contributors to mycotoxin contamination outbreaks. Two modeling techniques (GBM and NN) were applied. GBM analysis revealed that pre-planting temperature and precipitation were significant predictors of elevated AF and FUM levels. Additional key factors identified included soil characteristics, the normalized difference vegetation index (NDVI), and year-specific mean weekly temperature and precipitation. The NN models demonstrated strong class-specific predictive capabilities for one-year ahead validation, achieving accuracies of 73% and 85% for AF and FUMs, respectively. Therefore, the use of NN models is recommended for the annual prediction of mycotoxin contamination in maize [281].

Given the growing volume of research focused on AI applications for controlling toxigenic fungal species and detecting mycotoxins in food products and agricultural commodities, significant advancements are anticipated in this field in the coming years.

## 10. Conclusions

Mycotoxins remain a serious threat to human and animal health. This review outlines recent approaches for controlling toxigenic fungi and their mycotoxins, with growing interest in natural and technological alternatives to synthetic fungicides.

Essential oils offer a natural alternative with antifungal potential influenced by their active compounds, which depend on environmental and processing factors. While their antifungal mechanisms are known, their role in inhibiting mycotoxin production is less clear. Industrial application remains limited by sensory and toxicity issues.

Plant-derived polyphenols also show promise in reducing mycotoxin production in vitro, aided by antioxidant effects and gene expression modulation. However, regulatory restrictions and safety data gaps limit their current use.

Lactic acid bacteria (LAB), widely used in food preservation, have demonstrated the ability to inhibit both fungal growth and mycotoxin production, largely through the release of organic acids and other metabolites. Some LAB strains also reduce mycotoxin levels through adsorption or via antifungal compounds in cell-free broths.

Cold plasma technology is gaining attention as a rapid, cost-effective, and eco-friendly decontamination method. It works by generating reactive species that degrade mycotoxins and inhibit fungal growth. Despite its potential, standardization and evaluation of its effects on food quality are needed before industrial use.

Nanotechnology, especially the use of metal nanoparticles, has proven effective against fungal cells. Their efficacy depends on size, shape, and metal type, with smaller particles showing greater toxicity. However, safety concerns in human and animal health must be addressed before food industry application.

Magnetic nanocomposites, often enzyme-functionalized, have shown potential in adsorbing or degrading mycotoxins in liquids and can be easily removed with magnets, offering a practical detoxification tool.

Ionizing radiation remains a viable preservation technique, reducing fungal growth and mycotoxin levels. However, its broader use requires further research into its effects on nutritional quality and long-term safety.

Artificial intelligence, particularly ML and DL, is transforming mycotoxin prediction and control. These models have accurately forecasted fungal growth and mycotoxin synthesis in vitro and have been applied to classify contaminated foods and fields. The integration of hyperspectral and aerial imaging has enhanced these tools’ precision and applicability in real-time monitoring.

## 11. Future Perspectives

The strategies discussed in this review hold significant promise for preventing and controlling toxigenic fungi and mycotoxins at various stages of the food chain. These approaches can be applied individually or in combination. However, several aspects require further research before practical implementation. Research aimed at mitigating the negative effects and enhancing the positive attributes of these strategies is rapidly evolving. The primary challenges to be addressed in future research for each strategy are outlined below:

Essential oils (EOs): Future studies should delve deeper into aspects such as the following: (a) nanoencapsulation and active packaging incorporating EOs in nanocarriers like liposomes, nanoemulsions, cyclodextrins, nanoparticles, or biodegradable films/coatings to promote sustained release and reduce sensory impact; (b) synergistic combinations of EOs with synthetic antifungals or natural agents to lower EO concentrations, costs, and sensory rejection risks; (c) molecular targets and mechanisms using advanced technologies (transcriptomics, proteomics) to elucidate how EOs alter fungal cell membranes, inhibit toxin biosynthesis pathways, or affect fungal quorum sensing and spore germination; (d) applications in real food matrices (e.g., bread, cheese, fruits, nuts, and cereals) to evaluate the effects of EOs on food quality, nutrient preservation, and consumer perception; and (e) metabolomics to track how EOs reduce specific mycotoxins in food chains, facilitating a better understanding of dose–response relationships (e.g., enzyme inhibition, the modulation of oxidative stress). Additional challenges include standardizing EO quality (variations in chemotypes), regulatory hurdles (GRAS status may be insufficient), cost and feasibility considerations, and the potential development of fungal resistance over the long term.

Phenolic compounds: Similar to EOs, phenolic compounds represent a promising and sustainable strategy for controlling fungi and mycotoxins in food, contributing to both safety and quality. Their natural origin and proven antifungal and mycotoxin-inhibiting properties make them attractive alternatives to chemical preservatives. A promising research possibility involves exploring synergistic effects between phenolic compounds and other natural antifungal agents, such as combining them with EOs or other plant-derived antimicrobials to enhance antifungal activity and provide broader protection. However, further research is necessary to optimize their use and address potential issues related to stability, bioavailability, and consumer acceptance, as flavors or odors may limit their acceptability in certain food products.

Cold plasma (CP): Existing studies demonstrate CP’s ability to disrupt fungal cells (e.g., membrane oxidation, protein and lipid damage, DNA/RNA degradation, and the inactivation of fungal spores and biofilms) and degrade mycotoxins (e.g., structural decomposition, ROS-mediated oxidation and chain cleavage, hydroxylation, and decarboxylation). Nonetheless, additional research is required to (a) optimize in situ applications in real foods (e.g., cereals, nuts, spices, dried fruits, and cheese); (b) integrate CP with other methods such as EOs or polyphenols (synergistic ROS generation), active packaging (plasma-treated films with antifungal activity), or biocontrol agents (e.g., LAB + CP to reduce microbial load and enhance safety), and (c) other challenges such as scalability, penetration limitations, potential toxic by-products, regulatory considerations, cost, technical complexity, and the development of AI-assisted smart plasma systems for optimizing food treatments.

Lactic acid bacteria (LAB): Further research is needed to deepen understanding of aspects such as the following: (a) multifunctional antifungal mechanisms (e.g., organic acids, cyclic dipeptides and fatty acids, hydrogen peroxide, reuterin, and proteinaceous compounds); (b) mycotoxin binding and biotransformation (e.g., cell wall adsorption, enzymatic degradation, and metabolic conversion to less toxic forms); (c) omics technologies for strain selection (identifying LAB strains with strong antifungal and antimycotoxin traits, understanding the gene regulation of antifungal metabolite production, optimizing fermentation conditions to enhance protective activity); (d) synergistic use with other control agents (e.g., EOs, phenolic compounds, yeasts, edible films for active packaging systems); (e) the use of probiotic LAB strains (e.g., food detoxification, gut-level binding of ingested mycotoxins), and (f) synthetic biology and CRISPR engineering (e.g., engineering LAB to express enzymes targeting specific mycotoxins, boosting the production of antifungal metabolites). Additional considerations include strain variability (not all LAB are effective), stability and efficacy across different food matrices, regulatory aspects (particularly for genetically modified strains), and the consumer sensory perception of fermented products.

Metal nanoparticles (MNPs): Further research is essential to precisely understand (a) mechanisms of action (e.g., multimodal mechanisms, targeting fungi and mycotoxins); (b) green synthesis using plant extracts, fungi, or bacteria (to reduce toxicity and environmental impact, enhance biocompatibility, stabilize MNP structures, and add synergistic antifungal activity from bioactive compounds in plant extracts); (c) nanoformulation strategies to improve efficacy and minimize toxicity (e.g., nanocomposites combining MNPs with polymers or other nanoparticles, layer-by-layer coatings for controlled release, hybrid systems like MNPs + EOs or LAB); (d) integration into food systems (e.g., active food packaging films infused with MNPs to inhibit surface mold and mycotoxin production, grain storage systems with MNP sprays or sachets to reduce fungal load), and (e) toxicological and regulatory considerations (e.g., assessing cytotoxicity and bioaccumulation in food and human systems, developing regulatory frameworks for MNP use in food contact materials and direct applications, surface modification, and the coating of MNPs to reduce reactivity and toxicity, especially for AgNPs). Other challenges include addressing long-term toxicity and environmental impact, scaling up green synthesis methods, studying potential resistance development in fungi, the consumer acceptance of nanomaterials in foods, and establishing clear regulatory guidelines.

Magnetic materials: Emerging perspectives on using magnetic materials for controlling fungi and mycotoxins focus on aspects such as (a) antifungal activity (e.g., antifungal mechanisms, surface functionalization), the magnetic adsorption of mycotoxins (e.g., removing mycotoxins from various food matrices, including grains, nuts, and dried fruits); (b) high selectivity (e.g., modifying the surface of magnetic nanoparticles with specific ligands or antibodies selective for particular mycotoxins); (c) magnetic-functionalized food packaging (e.g., providing antimicrobial properties and controlling environmental conditions), magnetic field-enhanced fungal control (e.g., magnetic forces interfering with fungal biological processes like cell division or spore germination), and (d) biocompatibility and environmental safety (e.g., growing demand for natural, non-toxic, and eco-friendly food preservation technologies). Additional challenges involve scalability, regulatory approval, and consumer acceptance. In summary, further advancements in magnetic material design, surface functionalization, and application techniques are likely to yield more effective and sustainable solutions for combating fungi and mycotoxins in food.

Irradiation: Some of the new perspectives on the use of irradiation in controlling fungi and mycotoxins in food focus on several key aspects. These include (a) fungal control mechanism (e.g., action against a broad spectrum of toxigenic fungi, reduced spoilage, and extended shelf life), and (b) the reduction in mycotoxin contamination (e.g., the degradation of mycotoxins, the dose-dependent reduction in specific toxins. This is particularly relevant for commodities such as cereals, grains, nuts, and dried fruits, which are especially prone to fungal contamination; attention should be given to the safety and sensory impacts of irradiation on food quality, including attributes such as taste, texture, nutritional value, and overall consumer acceptance. The use of irradiation for decontaminating both raw and processed foods has the potential to improve food safety and quality by minimizing spoilage and post-processing mycotoxin production. Additionally, integrating irradiation with other preservation methods may produce synergistic effects, reducing the need for chemical preservatives. Key regulatory and safety considerations include approved irradiation doses, labeling requirements, and environmental or ethical concerns. Optimizing radiation doses for various food matrices is essential to maximize the reduction in fungal contamination and mycotoxins while preserving food quality. Finally, consumer education on the safety and benefits of irradiation, supported by transparent labeling, may help increase public acceptance and facilitate broader adoption of this technology in the food industry.

Artificial intelligence methods: Artificial intelligence (AI), particularly ML and DL, is poised to play an increasingly transformative role in the detection, prediction, and mitigation of toxigenic fungi and mycotoxins in food systems. With ongoing advances in data availability, sensor technologies, and computational power, future AI models are expected to become more accurate, adaptive, and capable of real-time decision making. Integration of AI with Internet of Things platforms and remote sensing technologies could enable the continuous monitoring of crops and storage environments, enhancing early warning systems. Moreover, explainable AI approaches may improve transparency and trust, facilitating wider adoption in regulatory frameworks and industrial applications. Continued interdisciplinary collaboration will be essential to fully realize AI’s potential in ensuring global food safety and mitigating mycotoxin-related risks.

## Figures and Tables

**Table 1:** Main mycotoxins and mycotoxin-producing fungi.

Mycotoxins	Main Producing Fungal Species
Aflatoxins B_1_ (AFB1) and B_2_ (AFB2)	*Aspergillus flavus*
	*A. parasiticus*
Aflatoxins G_1_ (AFG1) and G_2_ (AFG2)	*Aspergillus parasiticus*
	*A. flavus*
Ochratoxin A (OTA)	*Aspergillus welwitschiae*
	*A. westerdijkiae*
	*A. steynii*
	*A. carbonarius*
	*A. ochraceus*
	*Penicillium verrucosum*
	*P. nordicum*
Patulin (PAT)	*Penicillium expansum*
	*P. setosum*
	*Aspergillus* spp.
	*Byssochlamys* spp.
Type-A trichothecenes: T-2 toxin, HT-2 toxin, Diacetoxyscirpenol (DAS)	*Fusarium sporotrichioides* *F. langesethiae* *F. acuminatum* *F. poae*
Type-B trichothecenes:	
Deoxynivalenol (DON),	*Fusarium graminearum*
Nivalenol (NIV),	*F. culmorum*
3- and 15-Acetyldeoxynivalenol (3- and 15-ADON)	*F. poae* (NIV)
Zearalenone (ZEA)	*Fusarium graminearum* *F. culmorum*
Citrinin (CIT)	*Penicillium* spp.
	*Aspergillus* spp.
	*Monascus* spp.
Fumonisins (FUMs):	
Fumonisin B_1_ (FB1), Fumonisin B_2_ (FB2), Fumonisin B_3_ (FB3),	*Fusarium proliferatum* *F. verticillioides*
Other FUMs:	
Fusaproliferin	*Fusarium proliferatum*
	*F. verticillioides, Fusarium* spp.
Moniliformin	*Fusarium proliferatum*
	*Fusarium* spp.
Beauvericin	*Fusarium* spp., *A. niger*
Enniatins	*Fusarium avenaceum*
	*Fusarium* spp.
Ergot alkaloids	*Claviceps* spp.
Altenuene,	*Alternaria* spp.
Alternariol,	
Alternariol methyl ether,	
Altertoxin	
Tenuazonic acid	

## Data Availability

No new data were created or analyzed in this study. Data sharing is not applicable to this article.
